# Molecular Parallelism Underlies Convergent Highland Adaptation of Maize Landraces

**DOI:** 10.1093/molbev/msab119

**Published:** 2021-04-27

**Authors:** Li Wang, Emily B Josephs, Kristin M Lee, Lucas M Roberts, Rubén Rellán-Álvarez, Jeffrey Ross-Ibarra, Matthew B Hufford

**Affiliations:** 1 Shenzhen Branch, Guangdong Laboratory for Lingnan Modern Agriculture, Genome Analysis Laboratory of the Ministry of Agriculture, Agricultural Genomics Institute at Shenzhen, Chinese Academy of Agricultural Sciences, Shenzhen, China; 2 Department of Ecology, Evolution, and Organismal Biology, Iowa State University, Ames, IA, USA; 3 Department of Evolution and Ecology, University of California, Davis, Davis, CA, USA; 4 The Ecology, Evolution, and Behavior Program, Michigan State University, East Lansing, MI, USA; 5 Department of Plant Biology, Michigan State University, East Lansing, MI, USA; 6 Langebio, Irapuato, Gto., Mexico; 7 Department of Molecular and Structural Biochemistry, North Carolina State University, Raleigh, NC, USA; 8 Genome Center and Center for Population Biology, University of California, Davis, Davis, CA, USA

**Keywords:** convergent adaptation, flowering time, polygenic adaptation, population branch statistic

## Abstract

Convergent phenotypic evolution provides some of the strongest evidence for adaptation. However, the extent to which recurrent phenotypic adaptation has arisen via parallelism at the molecular level remains unresolved, as does the evolutionary origin of alleles underlying such adaptation. Here, we investigate genetic mechanisms of convergent highland adaptation in maize landrace populations and evaluate the genetic sources of recurrently selected alleles. Population branch excess statistics reveal substantial evidence of parallel adaptation at the level of individual single-nucleotide polymorphism (SNPs), genes, and pathways in four independent highland maize populations. The majority of convergently selected SNPs originated via migration from a single population, most likely in the Mesoamerican highlands, while standing variation introduced by ancient gene flow was also a contributor. Polygenic adaptation analyses of quantitative traits reveal that alleles affecting flowering time are significantly associated with elevation, indicating the flowering time pathway was targeted by highland adaptation. In addition, repeatedly selected genes were significantly enriched in the flowering time pathway, indicating their significance in adapting to highland conditions. Overall, our study system represents a promising model to study convergent evolution in plants with potential applications to crop adaptation across environmental gradients.

## Introduction

Convergent adaptation of populations to similar environments provides compelling evidence that natural selection, not neutral processes, shape trait variation. Similar phenotypes often arise independently in multiple species or populations exposed to the same evolutionary pressure (Davies et al. 2018; Alves et al. 2019). For example, species from four orders of insects, spanning 300 million years of divergence, have independently evolved tolerance to toxic compounds in milkweed and dogbane plant species (Dobler et al. 2012; Zhen et al. 2012). Likewise, reduction-of-function alleles at the *FLC* locus have evolved multiple times independently in *Capsella rubella* populations, conferring variation in flowering time (Yang et al. 2018). Pool et al. (2017) investigated cold adaptation across three pairs of highland and lowland *Drosophila melanogaster* populations, finding strong evidence for alleles that were repeatedly selected during highland colonization.

But while convergent phenotypes have been observed across the tree of life, in many cases their underlying genetic basis (e.g., molecular parallelism in which the same nucleotides, genes, or pathways are targeted versus convergence through independent molecular means) is unknown (Rosenblum et al. 2014; Storz 2016). Study of the genetics of convergence can help shed light on fundamental questions in evolutionary biology, including whether natural selection is constrained and repeatable or instead characterized by many molecular paths to similar phenotypes. The answer to these questions may depend, to a certain extent, on the trait itself. For example, the genetic architecture of a phenotype is a primary determinant of whether trait convergence results from parallel selection on orthologous loci (Yeaman et al. 2018). Such convergence is more likely for simple traits where only a few loci contribute or when loci are subject to antagonistic pleiotropy, and less likely for highly polygenic traits such as biomass or height (Orr 2005; Rosenblum et al. 2014; Yeaman et al. 2018).

Convergence through molecular parallelism, when it occurs, can originate in a number of ways. Genetic variation underlying convergent traits can arise as independent mutations, be derived from existing standing variation in a shared ancestral population, or be transferred between populations via migration (Lee and Coop 2017). Empirical studies have documented each of these modes of parallel adaptation. For instance, independent evolution of C4 photosynthesis in grasses (Christin et al. 2007) and sedges (Besnard et al. 2009) has involved multiple de novo mutations in the phosphoenolpyruvate carboxylase gene, a key C4 enzyme. In contrast, parallel adaptation in rabbit populations resistant to the myxoma virus in Australia, France, and the United Kingdom is achieved through selection on standing variation in immunity-related genes (Alves et al. 2019). Finally, convergence in patterns of wing coloration in butterfly species, an example of Müllerian mimicry, has resulted from gene flow across species (Dasmahapatra et al. 2012; Edelman et al. 2019).

Given its history, maize is an ideal model system to study parallel adaptation. Maize was first domesticated in the warm lowlands of the Balsas River Valley approximately 9,000 years ago (Matsuoka et al. 2002; Piperno et al. 2007), and subsequently spread to several independent highland regions: the Mexican Central Plateau, the highlands of the southwestern United States and Guatemala, and the Andes (Piperno and Flannery 2001; Dickau et al. 2007; Merrill et al. 2009; Piperno et al. 2009; Grobman et al. 2012; Hufford et al. 2012; da Fonseca et al. 2015; Bush et al. 2016; Stephen Athens et al. 2016; Brandenburg et al. 2017; Emerson et al. 2020; Wang et al. 2020). Although highland regions colonized by maize are far from identical, commonalities include a shorter growing season, low temperature, low partial pressure of atmospheric gases, and high ultraviolet radiation (Korner 2007; Lomax et al. 2012). Highland individuals exhibit several traits that are thought to be adaptive under these conditions, including highly pigmented and hairy leaf sheaths (Lauter et al. 2004; Rodriguez-Zapata et al. 2021). Common garden experiments have also demonstrated that highland maize flowers substantially earlier than lowland material (Eagles and Lothrop 1994; Jiang et al. 1999; Rodriguez et al. 2006).

The genetic basis of highland phenotypes across these populations is largely unknown, however. Earlier population genetic analysis of maize in Mexico and the Andes identified a small percent of single-nucleotide polymorphism (SNPs) showing evidence of selection in both populations, but concluded that maize highland adaptation arose largely independently in the two populations (Takuno et al. 2015). One contributor to the differences between these populations has been gene flow from wild relatives: adaptive introgression from the wild congener teosinte (*Zea mays* ssp. *mexicana*, hereafter *mexicana*) is thought to have played an important role during highland adaptation in Mexico (Hufford et al. 2013; Calfee et al. 2021; Rodriguez-Zapata et al. 2021), but maize has no wild relatives in South America and simulations suggested that long-distance gene flow between the populations is unlikely (Takuno et al. 2015). Nonetheless, this earlier work was limited to two highland populations and relied on limited genotyping data. Subsequent investigation using whole genome sequencing, for example, has discovered alleles introgressed from *mexicana* in both the Guatemalan and southwestern U.S. highlands outside of the distribution of *mexicana* (Wang et al. 2017), suggesting that migration from the Mexican highlands may not be as implausible as previously thought.

Here, we evaluate evidence for trait convergence through parallel molecular means using resequencing data from four highland populations of maize (Southwestern United States, Mexico, Guatemala, and the Andes) and two lowland populations (Mexico and South America). We assess the prevalence of molecular parallelism at the SNP, gene, and pathway levels, comparing models of de novo mutation, standing variation, and gene flow. Finally, our analysis of parallelism in pathways focuses on the well-characterized flowering time genes in maize and assesses signatures of polygenic adaptation for this trait.

## Results

### Description of Samples and Data

**Fig. 1.**
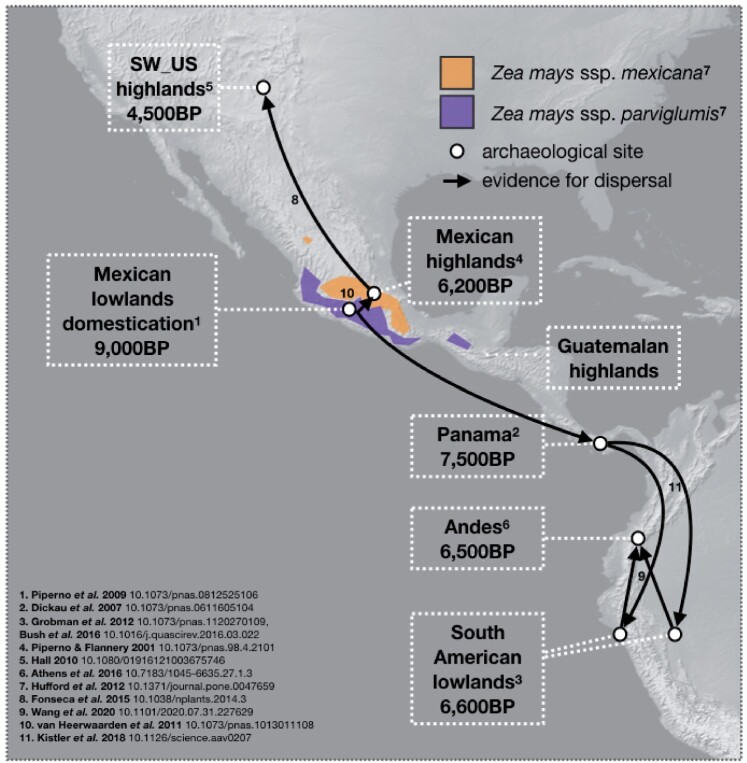
Sampling locations and expansion route of maize landraces, modified from Ross-Ibarra and Piperno (2020). Domestication and expansion times for maize populations are from published articles (Piperno and Flannery 2001; Dickau et al. 2007; Piperno et al. 2009; Hall 2010; van Heerwaarden et al. 2011; Grobman et al. 2012; Hufford et al. 2012; da Fonseca et al. 2015; Bush et al. 2016; Stephen Athens et al. 2016; Kistler et al. 2018; Wang et al. 2020). The exact location of our samples is indicated in supplementary fig. S1, Supplementary Material online.

Four independent highland populations (the Southwestern United States [hereafter referred to as US, six samples], Mexico [MH, six samples], Guatemala [GH, three samples], and the Andes [AN, five samples]) and two reference lowland populations (Mexico [ML, five samples) and South America [SL, six samples]) were sampled to investigate highland adaptation in maize landraces (fig. 1 and supplementary table S1, Supplementary Material online). SNPs called from high-depth, whole-genome resequencing data were obtained from our previous study (Wang et al. 2017). After filtering (see Materials and Methods), a total of 1,567,351 SNPs across 35 samples were retained for further analyses. We also characterized how environmental factors varied across our highland and lowland populations. A principal component analysis (PCA) of 19 bioclim environmental variables (http://www.worldclim.org/) revealed that highland and lowland accessions were differentiated along PC1 (comprising 51.8% of variation); whereas PC2 (comprising 22.8% of variation) reflected latitudinal differences across highland populations (supplementary fig. S2, Supplementary Material online). By plotting the loadings from the 19 bioclim variables, we found PC1 was primarily polarized by temperature seasonality and PC2 by precipitation (supplementary fig. S2, Supplementary Material online).

### Convergence through Parallelism at the SNP Level

In order to detect loci targeted by selection in highland populations, we utilized the Population Branch Excess (*PBE*) statistic (Yassin et al. 2016), which characterizes changes in allele frequencies in a focal population relative to two independent “outgroup” populations (Pool et al. 2017). *PBE* values were calculated for each SNP site and the top 5% were considered outliers and potential targets of selection.

To gauge the extent of convergence across our highland populations at the SNP level, we evaluated whether shared *PBE* outliers in pairs of populations and larger groups were more common than expected by chance. Significant overlap was observed in all pairs of highland populations (p≈0, hypergeometric statistic test; supplementary tables S2 and S3, Supplementary Material online). The Mexican Highland population showed the most substantial overlap in outlier SNPs with the Guatemalan Highlands (13,461 overlapped SNPs among 53,944 putatively selected SNPs in the Mexican Highlands; 7.5-fold enrichment) and the Southwestern U.S. Highlands (10,884 overlapped SNPs among 53,944 putatively selected SNPs in the Mexican Highlands; 6-fold enrichment). Although significantly more than expected, shared outliers between the Andes and all other highland populations were not nearly as extensive (4,031–6,702 overlapped SNPs among 51,453 putatively selected SNPs in the Andes; between 2- and 4-fold enrichment), consistent with reduced parallelism at the SNP level between these isolated regions (supplementary table S2, Supplementary Material online). The intersection of outlier SNPs across all highland populations was also significantly enriched (supplementary fig. S3, Supplementary Material online). Enrichment of overlapping selected sites was also confirmed in a more stringent tail of the top 1% of *PBE* values (supplementary fig. S4, Supplementary Material online) and in a data set that was thinned to account for linkage disequilibrium (supplementary fig. S5, Supplementary Material online). Although we did observe significant enrichment for parallelism at the SNP level, it is important to note that the majority of putatively selected SNPs (from 59.7% to 68.1%) showed signatures of adaptation in a single highland population.

**Fig. 2.**
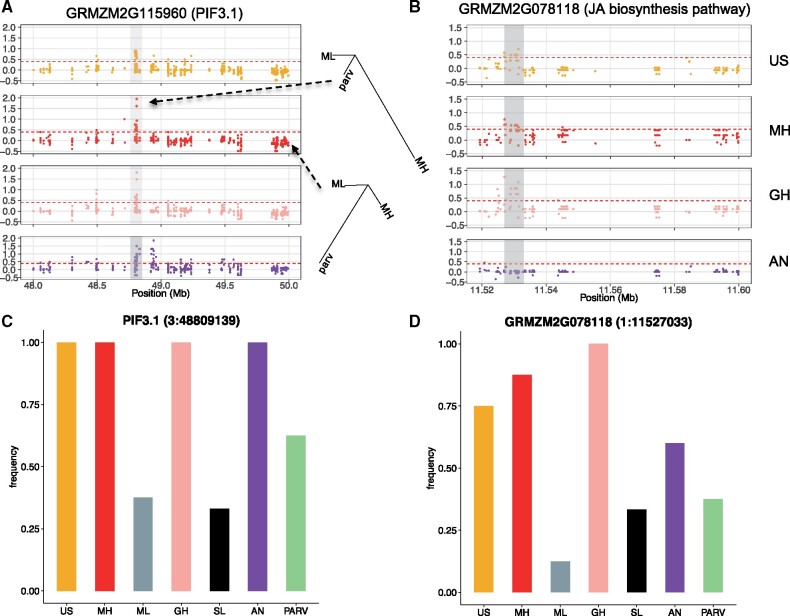
Patterns of parallel adaptation at two candidate loci. (*A*) Distribution of *PBE* values in a 2-Mb region around the gene *PIF3.1.* The branch length of inset trees was based on *PBE* values and indicates the difference between a selected and unselected SNP. (*B*) Distribution of *PBE* values in a 0.5-Mb region around the gene *GRMZM2G078118* involved in jasmonic acid biosynthesis. (*C*) Barplot of the reference allele frequency of one SNP located in *PIF3.1.* (*D*) Barplot of the nonreference allele frequency of one SNP located in *GRMZM2G078118*.

Our analysis of convergence at the SNP level using *PBE* revealed some particularly compelling candidate loci. For example, a SNP within *PIF3.1* (phytochrome-interacting factor) exhibited the highest *PBE* value in the Mexican Highland population and was also detected as a target of selection in all other highland populations (fig. 2*A*). A nonsynonymous, derived allele at this locus is fixed across all highland populations (fig. 2*C*). In addition, SNPs within *GRMZM2G078118*, a gene included in the jasmonic acid biosynthesis pathway, were detected as outliers in all Mesoamerican highland populations (Southwestern US, Mexican Central Plateau, Guatemalan ighlands), but not the Andes (fig. 2*B* and *D*).

**Fig. 3.**
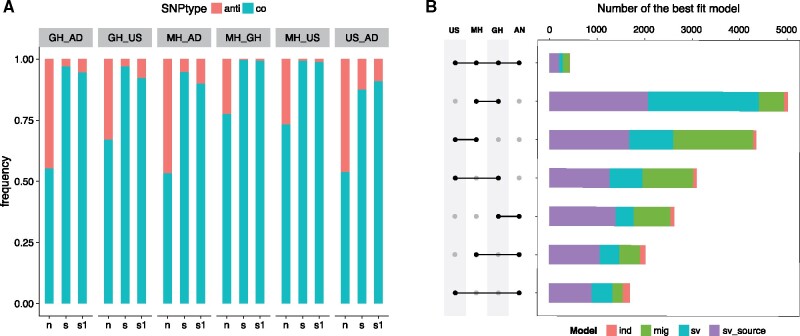
Patterns of parallel adaptation across loci. (*A*) Distribution of codirectional and antidirectional SNPs in neutral and outlier SNP sets. n: neutral SNPs; s: common outliers; s1: a subset of common outliers with MAF between 0.3 and 0.5. (*B*) Number of best fit models for genetic source of repeatedly selected SNPs. ind: independent de novo mutation; mig: migration; sv: standing variation; sv_source: standing variant with a source population. Abbreviations for populations: AN, Andes; GH, Guatemalan highlands; MH, Mexican highlands; US, Southwestern US highlands.

Although our *PBE* analysis reveals that selection targeted many of the same SNPs for adaptation across highland populations, this test alone does not clarify whether the same specific alleles were adaptive. We term outlier SNPs with the same allele elevated to high frequency in highland population pairs “codirectional,” and SNPs with different alleles as “antidirectional.” Predominantly, shared outliers in highland population pairs show codirectional allele frequency shifts (>95% in all population pairs with the exception of the Southwestern US/Andes comparison, which was 87.6%). These results provide stronger evidence of molecular parallelism at the SNP level (fig. 3*A*). In contrast, random samples of SNPs that do not show evidence of selection exhibit much reduced signals of codirectional change. Although random SNP samples between the Andes and other highland populations show roughly equal proportions of co- and antidirectionality, substantially more codirectional SNPs are observed in comparisons of Mesoamerican populations, perhaps reflecting their more closely shared histories (fig. 3*A*). However, the strong excess of codirectional changes in candidate SNPs may also be influenced by ancestral frequencies in lowland populations. For example, neutral drift has a greater likelihood of simultaneously fixing the same allele in multiple highland populations if the allele is already at high frequency in the lowlands. In order to address this bias, we first approximated the ancestral allele frequencies of outlier SNPs (based on the two-dimensional site frequency spectrum of Mexican lowland maize and the wild relative *Z. mays* ssp. *parviglumis*) in a subset of the neutral SNP set, and recalculated the number of codirectional and antidirectional SNPs. The same pattern was revealed (supplementary fig. S6, Supplementary Material online). Second, we subsampled outlier SNPs with a high minor allele frequency (i.e., between 0.3 and 0.5) and recalculated the ratio of codirectional versus antidirectional SNPs. We found antidirectional SNPs were slightly increased in this subset, but codirectional SNPs were still far more numerous (fig. 3*A*). Taken together, these results suggest that the observed codirectionality of allele frequency change in shared outlier SNPs provides strong evidence of molecular parallelism at the SNP level among highland maize populations.

### The Source of Shared Adaptation among Highland Populations

To determine whether shared *PBE* outlier SNPs had independent origins in highland populations, we applied DMC, a composite-likelihood-based inference method that distinguishes among possible mechanisms (Lee and Coop 2017; Tittes 2020). We controlled for possible effects of linkage disequilibrium by subsampling outliers to one SNP per 2-kb window. First, we examined SNPs selected in all four independent highland populations. We found the vast majority of these common outliers (421 among 428) had the highest composite likelihood under models where there was a single origin of the beneficial allele. Within this subset, 47.4%, 34.1%, and 16.8% of outliers had the standing variant model from a source population, the migration model and the standing variation model as best fits, respectively (fig. 3*B*). The standing variant model from a source population (outlined in detail in Appendix A.4 of Lee and Coop 2017) specifies that a beneficial allele originates in a single source population then spreads via ancient gene flow to other adapted populations where it may segregate for a standing time *t* prior to the onset of selection. Among shared outliers assigned the standing variant model from a source population, 70.9% and 25.6% had the second-most-likely model as the standing variation and migration models, respectively, with 82.3% of these showing likelihood value differences <1 between the best-fit and second-best-fit models. Considering the potential for overfitting with the standing variant model with a source population, which has four parameters compared with three parameters in the migration and standing variant models, we propose that the majority of common outliers assigned as the standing variant with a source model were virtually indistinguishable from the standing variant and migration models. For the common outliers identified under the recent migration model, the main migration sources were the Mexican (43.9%) and Guatemalan (41.9%) highland populations, a result that could be partially explained by the substantial shared ancestry of these two populations and direct maize colonization from the Mexican highlands to the highlands of both the Southwestern US and Guatemala. In summary, results for outlier SNPs shared across all four highland regions suggest gene flow among highland populations and standing variation may have been the major sources of adaptive variation.

We further explored the source of shared adaptive variation across pairwise samples of highland populations. As seen in four-way comparisons after correction, the standing variant with a source population model (38.5–52.9%; mean 46.5%), the standing variation model (14.5–46.4%; mean 24.0%), and the migration model (10.5–38.7%; mean 25.9%) were most commonly supported (fig. 3*B*). The standing variant with a source model, which includes an extra parameter, showed only a slightly higher likelihood than the simple standing variation and migration models, indicating these sources may also be likely drivers of convergence. It is noteworthy that the parallelism level among Mesoamerican highland populations was much stronger than observed between the Andes and other highland populations (fig. 3*B*). Not only do fewer loci show evidence of selection in pairs of populations including the Andes, but repeatedly selected loci are more likely to have arisen independently: 6.1% of shared loci were identified as having independent origins in Andean comparisons, in contrast to a mean of 1.9% within Mesoamerican highland population comparisons (fig. 3*B*).

Previous studies have suggested that migration between the Andes and Mesoamerican populations is unlikely (Takuno et al. 2015). We therefore further evaluated all repeatedly selected SNPs by calculating the divergence statistic, *F_ST_*, between pairwise highland populations to assess how this varies based on the assigned model. Repeatedly selected SNPs consistent with a migration model exhibited weaker divergence (lower *F_ST_*; p≈0; supplementary fig. S7, Supplementary Material online), an additional indicator of gene flow. We also calculated the $\hat{f_{dM}}$ statistic (Malinsky et al. 2021) in 10-*kb* nonoverlapping windows across the genome. In a (((P1, P2)P3),O) shaped phylogenetic tree, the $\hat{f_{dM}}$ statistic gives positive values for introgression between P3 and P2 and negative values for introgression between P3 and P1. We found SNPs consistent with the migration model demonstrated more positive $\hat{f_{dM}}$ values, providing additional support for gene flow between the Andes and Mesoamerican highland populations (supplementary fig. S8, Supplementary Material online). In summary, although the proportion of dually selected SNPs between the Andes and Mesoamerican highland populations (from 7.8% to 11.2%) was consistent with previous work (7–8%) (Takuno et al. 2015), the two most supported source models were standing variation and migration, suggesting a more prominent role for migration than previously hypothesized (Takuno et al. 2015).

### Molecular Parallelism at the Genic Level

We next evaluated the extent of molecular parallelism at the gene level. A gene was classified as an outlier if it contained *PBE* outlier SNPs within the gene or 10 kb upstream or downstream (supplementary table S4, Supplementary Material online). A total of 1,651 candidate genes, among the total of 29,160 genes with sufficient sequence depth to be tested, were observed in the Mexican Highlands, among which 360 (21.8%) contained or were near to outlier SNPs which were shared with at least one other highland population. The percentage dropped to 18.9% in the Guatemalan highlands, 10.0% in the Southwestern U.S. highlands and 9.4% in the Andes (supplementary table S5, Supplementary Material online). We utilized the hypergeometric test implemented in the R package SuperExactTest (Wang et al. 2018) to evaluate the two- to four-degree intersection of outlier genes among highland populations. As observed in results at the SNP level, significant overlap was observed for each comparison (supplementary figs. S9 and S10, Supplementary Material online). In order to account for the effects of linkage disequilibrium on selected genes, we thinned outlier SNPs with $r^{2}\leq 0.2$ within 10-kb windows and reevaluated the overlap of selected genes. This thinned set showed consistent results (Supplementary Material online), suggesting the pattern was not caused by clustered outlier genes in selective sweeps. In many instances, common outlier genes involved selection on different SNPs across highland populations. For example, 349 (21.1%) of the 1651 selected genes in the Mexican highlands were in common with other highland populations, but showed evidence of selection on distinct SNPs. The percentage was 18.8% in Guatemala, 24.7% in the Southwestern US, and 18.5% in the Andes (supplementary table S5, Supplementary Material online).

### Constraint of the Adaptation Target Size Leads to Molecular Parallelism

There are two explanations for the extent of molecular parallelism we have observed in genes selected in the highlands (Yeaman et al. 2018). First, it is possible that adaptation is constrained by the number of genes that can affect a trait, effectively placing physiological limits on the routes adaptation can take. Alternatively, many different genes may have the potential to affect the trait, but deleterious pleiotropic effects of variation in some genes may prevent them from playing a role in adaptation. We will refer to these possibilities as “physiological” and “pleiotropic” constraint. Consistent with our analysis above, Yeaman et al. (2018)’s $C_{\chi^{2}}$ statistic finds strong evidence that selection at the same loci is shared among populations more often than can be explained by chance ($C_{\chi^{2}}$ from 19.6 to 40.1 with a mean of 28.05, permutation *P *<* *0.0001). To further evaluate constraint in parallel adaptation, we tested an alternative null model which assumes the set of genes selected in at least one population represents all genes that can possibly affect the trait. We then tested whether there was evidence of excessive sharing beyond the expectations under physiological constraint (Yeaman et al. 2018). Here, we found that $C_{\chi^{2}}$ was less than 0 in all highland pairs except the Mexican and Guatemalan highlands pair ($C_{\chi^{2}}=1.9,\;P=0.025$), indicating sharing beyond that expected under physiological constraint and consistent with the known substantial shared ancestry between these two highland populations. These results indicate that repeated selection of genes among most highland pairs may simply be due to physiological constraint in the number of loci that can contribute to variation in a trait. To explore this possibility further, we assessed the prevalence of molecular parallelism in the flowering time pathway, a well-characterized gene network known to be of adaptive importance in highland environments.

### Molecular Parallelism within the Flowering Time Pathway

**Fig. 4.**
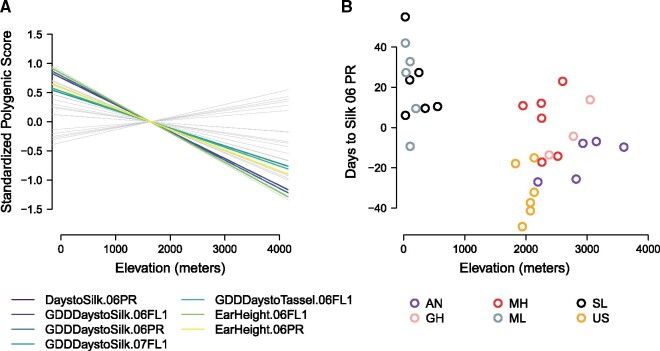
Polygenic adaptation along elevation in the landraces. (*A*) A linear regression between polygenic score for all 29 trait-environment combinations tested and elevation of origin. The lines for the seven traits that showed significant signals of polygenic adaptation are colored and all other traits are shown in gray. (*B*) Polygenic score for days to silk in Florida (2006) for all landraces is negatively correlated with elevation of origin.

Flowering time in maize is known to be a highly quantitative trait and an example of polygenic adaptation (Buckler et al. 2009; Navarro et al. 2017; Gates et al. 2019; Josephs et al. 2019). As such, we may expect that highland adaptation for flowering is the result of subtle, coordinated allele frequency shifts across many loci. In evaluating flowering time genes we tested: 1) the strength of selection on loci associated with flowering time relative to expectations based on drift, and 2) the intersection of selected genes in the pathway in multiple highland regions. To first identify whether flowering time was targeted during highland adaptation, we conducted a genome-wide association study (GWAS) of 29 traits measured in short-day conditions in Ponce, Puerto Rico and Homestead, Florida (Hung et al. 2012) using a maize association mapping panel comprised of tropical and temperate inbred lines (Flint-Garcia et al. 2005). We then summarized the frequency of multiple alleles affecting a trait of interest with a polygenic score and tested for associations between polygenic score and elevation of origin of our landraces, while controlling for variation in relatedness between samples following Josephs et al. (2019). A significant result implies that allele frequencies that increase trait value are more common at one end of the elevation gradient (either high or low) than expected due to drift. Seven traits showed a significant relationship between their conditional polygenic score and elevation with a false discovery rate < 0.1. These traits were primarily related to flowering time: Days to Silk, Growing Degree Days to Silk, and Ear Height from ’06 Puerto Rico, Growing Degree Days to Silk, Growing Degree Days to Tassel, and Ear Height in ’06 Florida, and Growing Degree Days to Silk in ’07 Florida (fig. 4).

**Fig. 5.**
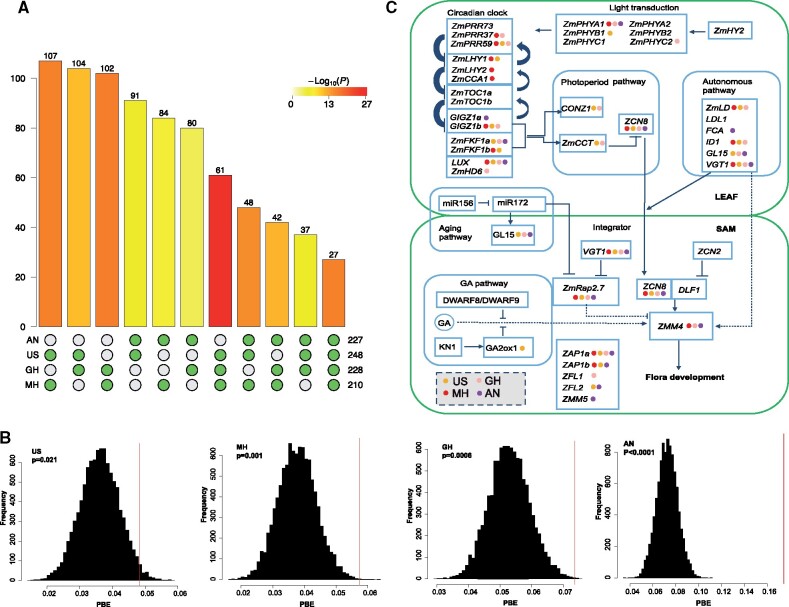
Convergent adaptation in the flowering time pathway. (*A*) Intersection of flowering time outlier genes among four highland populations. (*B*) Distribution of mean *PBE* values of SNPs located within and in the 10-kb flanking regions of core flowering time genes (Dong et al. 2012) (the red line) against a background of all genes (the black bars). (*C*) Diagram showing selected genes in the flowering time network. Colored dots indicate the population(s) in which selection was detected.

Given this support for selection on flowering time during highland adaptation, we explored the overlap between our selected genes and known flowering time candidates. First, we utilized a list of 904 maize flowering time candidate genes aggregated by Li et al. (2016) (supplementary table S6, Supplementary Material online). Within this list, we found a substantial excess of flowering time genes targeted by selection among groups of two, three, and four highland populations (p<2.87e−5, hypergeometric test; fig. 5*A* and supplementary table S7, Supplementary Material online). Outlier SNPs within these genes also showed strong codirectional changes in allele frequency across highland populations (91.8−100%) (supplementary fig. S11, Supplementary Material online). As observed in our genome-wide analysis, candidate sharing at flowering time loci was stronger among Mesoamerican highland populations than between comparisons with the Andes (fig. 5). We also applied the $C_{\chi^{2}}$ statistic to this list of genes and confirmed enriched sharing of selected genes among highland populations (mean $C_{\chi^{2}}$ 6.618, permutation *P *<* *0.0001). Our finding of an excess of shared candidates across regions within a large set of known genes that affect flowering time suggests physiological constraint alone cannot explain patterns of convergence, but instead that variation at only a subset of genes can affect flowering without deleterious pleiotropic consequences.

We then narrowed our focus to a subset of core flowering time genes (Dong et al. 2012) with highly characterized functional roles (fig. 5*C*) based on published QTL or GWAS analyses. Thirty-two of the 39 core flowering time genes (contained at least one outlier SNP within the gene or in the 10-kb flanking regions) in at least one highland population, which indicated overrepresentation of flowering time genes among all selected genes (*P *=* *0.005, hypergeometric test). Although common outlier genes between the Andes and other highland populations were not significantly enriched in this smaller list (*p *>* *0.074, hypergeometric test; supplementary table S7, Supplementary Material online), enrichment was observed among all other two and three highland population comparisons (*P *<* *0.042, hypergeometric test; supplementary table S4, Supplementary Material online). These results were confirmed through calculation of the $C_{\chi^{2}}$ statistic (Mesoamerican highland population comparisons, mean $C_{\chi^{2}}$ 2.32, permutation *P *<* *0.01; comparisons to the Andes, mean $C_{\chi^{2}}$ 1.575, permutation *P* from 0.015 to 0.054).

The *PBE* values of outlier SNPs within or in the 10-kb flanking regions of flowering time genes were in the extreme tail of the distribution of all genes (fig. 5*B*), with some of the best-characterized flowering time genes in maize clearly targeted by selection in multiple highland populations (fig. 5*C*). For example, *ZCN8* (*GRMZM2G179264*), which is orthologous to *FLOWERING LOCUS T* (*FT*) in *Arabidopsis*, has been shown to play a significant role in maize adaptation to high latitudes (Guo et al. 2018) and was selected in all four highland populations in our study. Floral transition is regulated by several MADS-box genes, particularly *ZMM4* (*GRMZM2G032339*), which is under selection in all but the Southwestern U.S. highlands. *VGT1* (*GRMZM2G700665*; vegetative to generative transition 1) was identified as an outlier in all four highland populations, with the same nonsynonymous SNP (8:131578990, indicating locus 131578990 on chromosome 8) targeted in the Mexican and Guatemalan highlands (supplementary fig. S12, Supplementary Material online). In contrast, in the Southwestern U.S. highlands, the selected SNP (8:131580179) in *VGT1* was located in the 3′ UTR region and, in the Andes, the selected SNP (8:131579463) was a synonymous mutation near the 3′ UTR (supplementary fig. S12*A*–*C*, Supplementary Material online). Two *CONSTANS* (*CO*) genes, *CONZ1* and *ZmCCT*, were selected in both the Mexican and Guatemalan highland populations. We further found *Gigantea2* (*GRMZM5G844173*) was selected in all but the Guatemalan highlands and *FKF2* (*GRMZM2G106363*) in all but the Mexican highlands. Among circadian clock genes, *LUX* (*GRMZM2G067702*) (supplementary fig. S12*D*, Supplementary Material online) was selected in all four highland populations and *ZmPRR59* was an outlier in the three Mesoamerican highland populations. In addition, the F box protein *ZEITLUPE* (*GRMZM2G113244*), *RVE2* (*GRMZM2G145041*) and *PRR5* (*GRMZM2G179024*) were selected in all four highland populations. Finally, the light transduction gene *PHYA1* (*GRMZM2G157727*) was targeted by selection in all but the Southwestern U.S. highland populations.

## Discussion

The prevalence of parallel evolution is a long-standing question in the field of evolution. Through a comprehensive evaluation of parallel adaptation among independent maize highland populations, we found that, whereas most adaptation is independent, parallelism is more common than expected. We observed a particularly high level of parallelism among Mesoamerican highland populations relative to comparisons including Andean maize. The vast majority of parallel adaptive alleles we discovered have risen in frequency in the highlands from migration and standing variation, and only a small percentage represent de novo mutations. However, the proportion of repeatedly selected SNPs from de novo mutations is the highest in pairs of populations that include the Andes. Our analyses further reveal that adaptive routes from genotype to phenotype have been canalized by both physiological (i.e., few genes contributing to a trait) and pleiotropic (i.e., a subset of causal genes can contribute to trait variation without negative pleiotropic consequences) processes. For example, we observe that only a subset of possible genes related to flowering time contribute to highland adaptation, with many of these loci showing evidence of selection in multiple highland regions, particularly in Mesoamerica.

Approximately one quarter (mean 24.0%) of highland adaptation SNPs showed a pattern of molecular parallelism in Mesoamerica. A smaller percentage also showed this pattern in Andean comparisons (mean 10.7%), but this represents a slightly higher proportion than previously reported (7–8% between the Andes and the Mexican Highlands) (Takuno et al. 2015). We have also expanded upon previous studies of parallel highland maize adaptation (Takuno et al. 2015) in a number of ways. Our study has broadened the geographic scope of sampling for highland maize populations and is the first such study using high-depth, whole-genome resequencing data, which allowed us to attain a more complete picture of parallelism. Additionally, our use of the *PBE* statistic allowed for detection of selection specifically in the highlands and reduced the extent of false positives, a limitation of studies that focus solely on population differentiation outliers. Our polygenic adaptation analyses also helped to clarify specific phenotypes targeted by selection during adaptation, showing clear evidence of selection on flowering time. We have also uncovered a pervasive role of migration in transferring adaptive alleles across highland regions. In fact, our analyses revealed a moderate proportion of parallel adaptation through migration even between the Andes and other highland populations, in contrast to previous studies that appeared to exclude this possibility based on a simple population genetic model (Takuno et al. 2015). This finding is consistent with a recent study which showed that some traits, for example flowering time, may not demonstrate a GxE effect on fitness (Gates et al. 2019), allowing adaptive alleles to move between regions through a matrix of habitat (e.g., the lowlands) where they do not clearly confer an adaptive advantage.

The extensive parallelism in maize highland adaptation has likely been affected by multiple factors and is consistent with expectations based on population genetic theory. First, a high level of genetic diversity in the species provides substantial standing genetic variation and potentially beneficial mutations; indeed, Zhao and Begun (2017) showed that the more genetically diverse species *Drosophila hydei* contained more adaptive alleles than *D. melanogaster*. Second, the recent timing of expansion and divergence in maize has likely affected the extent of parallelism. The probability of parallelism will decrease when populations diverge over a greater period of time, as less shared standing variation would be expected to be the source of adaptation. Increased divergence time would also allow for novel beneficial mutations to arise, potentially in distinct genes and pathways (Conte et al. 2012). For example, Preite et al. (2019) demonstrated a moderate level of molecular parallelism in adaptation to calamine metalliferous soils within *Arabidopsis* species, but less parallelism between species. In addition, a recent study (Bohutinska et al. 2020) discovered that the degree of molecular parallelism decreases with increasing divergence between lineages in alpine *Arabidopsis* species. Third, parallel adaptation from standing genetic variation is unlikely at a large number of loci (MacPherson and Nuismer 2017), highlighting the important role of physiological constraint—which limits the number of loci at which variation can affect a trait—in determining patterns of convergence. Fourth, population genetic modeling predicts that traits with pleiotropic constraint are more likely to demonstrate molecular parallelism (Yeaman 2015). Despite the quantitative nature of flowering time in maize, the $C_{\chi^{2}}$ statistic still showed evidence of genetic constraint and convergence, even after limiting our null to genes in the known flowering time pathway. Similarly, a recent study found molecular parallelism for cold tolerance between distantly related species (lodgepole pine and interior spruce), indicating certain key genes playing crucial roles in cold adaptation (Yeaman et al. 2016). Finally, theory predicts that gene flow may have contrasting impacts on parallel adaptation. Gene flow between differentially adapted populations may introduce maladaptive alleles, counteracting the effects of local selection (Yeaman 2015). For example, Holliday et al. (2016) found islands of divergence in *Populus trichocarpa* populations across altitudinal clines and proposed that coadapted genes in strong linkage may buffer against genetic introgression from maladapted individuals. Our results, consistent with earlier studies (Hufford et al. 2013; Takuno et al. 2015), suggest limited gene flow into the highlands from maladapted lowland maize. Instead, we observe, as a driver of adaptation and parallelism, an important role for adaptive introgression into maize from a highland wild relative and spread of these alleles through gene flow to nearby highland regions. Similarly, adaptive introgression at the EPAS1 locus has been shown to underlie convergent highland adaptation in both humans (Huerta-Sánchez et al. 2014) and dogs (Li et al. 2014).

In summary, the emerging story of parallel highland adaptation in maize is consistent with existing theory. Gene flow is common across maize populations but does not appear to swamp locally adaptive alleles. Rather, highland adaptation appears to have initially been facilitated in the Central Plateau of Mexico by introgression from the locally adapted wild species, *mexicana* (Hufford et al. 2013; Calfee et al. 2021; Rodriguez-Zapata et al. 2021). Subsequently, gene flow from the Mexican highlands appears to have contributed to highland adaptation in other regions of the Americas (Wang et al. 2017), consistent with our finding of migration as a major source of molecular parallelism. Novel alleles from *mexicana* augmented the extensive standing variation already found within the species (Hufford et al. 2012), providing a pool of adaptive variants that appear to have independently risen in frequency in multiple highland regions.

Similar adaptive processes (e.g., gene flow with newly encountered, locally adapted populations and species, repeated selection on standing variation in independent but similar habitats) likely occurred as other crops expanded from their centers of origin (Janzen et al. 2019). However, crops that were domesticated from wild species with small effective population sizes, with more pronounced domestication bottlenecks, or without widespread, locally adapted congeners may have lacked the adaptive potential necessary to achieve the broad and varied distribution of maize. The success of invasive species has also been linked to hybridization (Heim et al. 2020) and high levels of standing variation (Mimura et al. 2013). A thorough understanding of adaptive processes during rapid expansion across diverse habitats may therefore inform invasive species mitigation strategies, assisted migration in the face of climate change, and crop breeding for tolerance of extreme environmental conditions. Highland adaptation in maize is a clear example in which adaptation has drawn substantially from a shared pool of variants, with the same alleles, genes, and pathways contributing to the evolutionary success of the species across continents and millennia.

## Materials and Methods

### Samples and Data

Samples and SNPs were from our previous publication (Wang et al. 2017) (supplementary table S1, Supplementary Material online). SNPs were removed when located in centromeric regions, and later filtered to retain only those with sequencing depth greater than 10× and minor allele frequency greater than 0.05 (supplementary fig. S13, Supplementary Material online). SNPs located within known large inversions and *mexicana* introgression regions (primarily in low-recombination regions such as pericentromeres) were removed from analyses due to nonindependence of SNPs within these regions and their enrichment for highly differentiated loci. The filtered SNP data set was shared through the CyVerse platform (https://de.cyverse.org/dl/d/54685BD0-ADCF-4796-8634-12388B097060/wang2020genotype.tar.gz; last accessed April 29, 2021). Nineteen environmental variables were obtained from the WorldClim data set (http://www.worldclim.org/; last accessed April 29, 2021) and were utilized to perform a PCA with the vegan package in R (Oksanen et al. 2016). We employed variant effect predictor from Ensembl (McLaren et al. 2016) to further annotate genomic location and functional consequences of SNPs.

### *PBS* and *PBE* Calculation

Compared with the commonly used statistic to estimate levels of population differentiation—*F_ST_*, *PBS* calculates allele frequency change specific to one focal population by adding pairwise *F_ST_* values involving a third “outgroup” population (Yi et al. 2010).

(1) $$\mathrm{PBS}=\frac{T^{HL_{1}}+T^{HL_{2}}-T^{L_{1}L_{2}}}{2}$$

(2) $$T=-\log(1-F_{ST})$$

*PBE* is a recently described derivative of *PBS*, which overcomes the limitation of *PBS* values being high when all populations have long branches. *PBE* instead quantifies the difference of the observed and the expected *PBS* values (Yassin et al. 2016).

(3) $$\mathrm{PBE}=PBS_{\mathrm{obs}}-PBS_{\;\text{exp}\;}=\mathrm{PBS}-\left[T^{L_{1}L_{2}}*\frac{PBS_{\mathrm{med}}}{T_{\mathrm{med}}^{L_{1}L_{2}}}\right]$$

Here, $T^{L_{1}L_{2}}$ quantifies genetic differentiation of the two nonfocal populations. *PBE* is expected to be strongly positive when selection acts specifically on the focal population.

We calculated *PBE* for filtered SNPs among one highland population, its corresponding lowland population (the Mexican lowland population was paired with the Mesoamerican highland populations; the South American lowland population was paired with the Andes population) and the *parviglumis* Palmar Chico population (supplementary fig. S13, Supplementary Material online).

Pairwise Weir–Cockerham *F_ST_* values were calculated in vcfTools (Danecek et al. 2011) and custom R scripts were used to compute *PBS* and *PBE* values of the focal highland population. SNPs with *PBE* values higher than the 95% quantile of its distribution were regarded as outliers and qualitative results were confirmed in the 99% quantile. The R package “SuperExactTest” (Wang et al. 2018) was utilized to evaluate if the overlap of outlier SNPs between pairwise highland populations was enriched. In order to address false detection of overlap driven by linkage disequilibrium among loci, we removed loci with an *r*^2^ greater than 0.2 within 10kb windows and re-evaluated the significance of overlap among highland populations.

In addition, we also evaluated if the same allele is elevated to high frequency among outlier SNPs between pairwise highland populations (supplementary fig. S14, Supplementary Material online). If there is no allele frequency change between the highland and its lowland counterpart, we categorized the SNPs as “nondirectional.” When a SNP was nondirectional in both highland populations being compared, it was removed from the analyses. When the same allele was at elevated frequency in both highland populations compared with their lowland counterparts, the SNP was categorized as “codirectional”. When different alleles were at high frequency across two highland populations, the SNP was categorized as “antidirectional”. We then compared the proportion of co-directional SNPs between the outlier and neutral SNPs.

In order to exclude the bias of genetic drift on the directionality of changes in allele frequency, we approximated the two-dimensional site frequency spectrum (2dSFS) of outlier SNPs using a subset of the neutral SNP set. We divided the nonreference allele frequency in Mexican Lowland maize and *parviglumis* into ten equal bins in both the outlier and neutral SNP set. Then we matched neutral SNPs with the same ancestral 2dSFS (representing the combination of allele frequencies in both the Mexican Lowland and *parviglumis* populations) with the outlier SNP set and sampled the same amount of SNPs in each allele frequency bin. Last, we tabulated the directionality of the randomly sampled neutral SNPs to check how ancestral allele frequency influenced the proportion of codirectional SNPs.

### Composite Likelihood Calculations to Distinguish the Mode of Parallelism

Following the method developed and applied in Lee and Coop (2017) and Tittes (2020), we calculated the composite likelihood under four models for the outlier regions shared among all four highland populations, after thinning for linkage. The four models tested were as follows: 1) independent mutations of the beneficial allele in four highland populations, 2) a single origin of the beneficial allele in one single highland population and spread via gene flow into the other population during the sweep, 3) standing variation present in the ancestor of the four highland populations, 4) gene flow contributing novel standing variation which, *t* generations later, is selected. The details of these four models can be found in Lee and Coop (2017). For each outlier, we obtained SNPs in the surrounding 20-kb window and calculated the composite likelihood under each of the four models for this data set. We compared composite likelihoods under the models for each outlier and obtained maximum composite-likelihood-parameter estimates. Similarly, we also evaluated the composite likelihood values under four models for each set of dually selected SNPs in pairs of highland populations.

### Constraint of the Adaptation Target Size

Following the method in Yeaman et al. (2018), we first based our analyses on a null hypothesis that all genes contribute equally to adaptation. Second, we set up a new null hypothesis that only the selected genes (those selected in at least one highland population) could contribute to adaptive traits. With those two different null hypotheses, we calculated $C_{\chi^{2}}$ and the corresponding *P* values and compared the differences.

### Polygenic Adaptation for Quantitative Traits

We followed the methods developed by Berg and Coop (2014) and Josephs (2018) to detect polygenic adaptation in the highland populations. Briefly, these methods detect adaptive divergence for a trait by 1) finding loci associated with that trait in a GWAS, 2) summarizing the allele frequencies and effect sizes at these loci in the populations of interest using a polygenic score, and 3) testing to see if the association between these polygenic scores and an environmental character (in this case, elevation) are greater than could be explained by drift.

To identify loci associated with traits that could be under selection, we conducted GWAS using a maize panel developed for GWAS, often referred to as “the 282” or “the Major Goodman panel” (Flint-Garcia et al. 2005). SNPs for 263 individuals in the Major Goodman panel were called from whole genome sequencing data from Bukowski et al. (2017), removing individuals with genotype calls for <70% of polymorphic sites. Of all the SNPs called in Bukowski et al. (2017), we only used those that were also polymorphic in the maize landraces and that had a MAF > 0.01 and were missing data for <5% of individuals, leaving us with approximately 5 million SNPs per test. We used trait measurements made in three short-day common garden experiments, from Florida in 2006 and 2007 and Puerto Rico in 2006, from Hung et al. (2012) where there were data available for at least 80% of the 263 individuals, leaving us with 29 trait-environment combinations. We conducted GWAS using GEMMA with default parameters (Zhou and Stephens 2012), with a standardized kinship matrix to control for population structure.

We used the GWAS hits to generate polygenic scores for each individual in the landrace panel using all loci with a *P* value < 0.1 that had been pruned down to the strongest hit per 1 cM window using a linkage map from Ogut et al. (2015), leaving us with an average of 216 SNPs per trait (range = 147–289). If there are *M* loci associated with a trait, each with effect size *α_m_*, *p_im_* is the allele frequency of the *m*th allele in line *i*, which will either be 0, 0.5, or 1, then we can calculate the polygenic score for the *i*th line as *Z_i_* where

(4) $$Z_{i}=2\sum_{m=1}^{M}\alpha_{m}p_{im}$$

Shared population structure between the GWAS panel and the test panel can lead to false positive signals of selection (Josephs 2018). To test for shared structure between the GWAS panel used here and the landraces, we constructed a joint kinship matrix between all samples in both panels, following the same methods used in Josephs (2018). Plotting the first two eigenvectors of this joint kinship matrix (the principal components) shows that there is likely shared population structure between the landraces and the GWAS panel (supplementary fig. S15*A*, Supplementary Material online). We constructed a conditional kinship matrix that estimates the amount of relatedness between landraces conditional on relatedness captured in the GWAS panel, and found that this new conditional matrix explains less variation than the original matrix made from landraces alone (supplementary fig. S15*B*, Supplementary Material online). This finding is further evidence that there is shared population structure between the two panels.

In light of this shared structure, we conducted a conditional test, following Josephs (2018). Let $\vec{Z_{1}}$ be the vector of *Z_i_* for the 31 highland and lowland landraces discussed in this paper and $\vec{Z_{2}}$ is the vector of *Z_i_* for the individuals used in the GWAS. We model the combined vector of polygenic scores in both panels as

(5) $$\left(\begin{array}{c} {\vec{Z}}_{1}\\ {\vec{Z}}_{2} \end{array}\right)∼\mathrm{MVN}\left(\left(\begin{array}{c} \mu \\ \mu \end{array}\right),V_{A}\left(\begin{array}{cc} K_{11} & K_{12}\\ K_{12}^{T} & K_{22} \end{array}\right)\right).$$

where, *μ* is the mean of the combined vector $\left[X_{1},X_{2}\right]$, *K*_11_ and *K*_22_ are the kinship matrices of the genotyping and GWAS panels, and *K*_12_ is the set of relatedness coefficients between lines in the genotyping panel (rows) and GWAS panel (columns).

The conditional multivariate null model for our polygenic scores in the landraces conditional on the GWAS panel is then

(6) $${\vec{Z}}_{1}|{\vec{Z}}_{2}∼\mathrm{MVN}(\vec{\mu }\prime ,2V_{A}K\prime ),$$

where $\vec{\mu }\prime$ is a vector of conditional means with an entry for each sample in the genotyping panel:

(7) $$\vec{\mu \prime }=\mu +K_{12}K_{22}^{-1}({\vec{Z}}_{2}-\mu )$$

and K′ is the relatedness matrix for the genotyping panel conditional on the matrix of the GWAS panel,

(8) $$K\prime =K_{11}-K_{12}K_{22}^{-1}K_{12}^{T}.$$

To test for selection, we calculate the difference between observed polygenic scores and conditional means as $\vec{Z}\prime =\vec{Z_{1}}-\vec{\mu \prime }$. Higher values of $\vec{Z}\prime$ indicate that the observed polygenic score in a landrace individual is greater than would be expected based on relatedness between that landrace individual and individuals in the GWAS panel.

After calculating $\vec{Z}\prime$ for 29 environment-trait combinations, we test for a correlation between $\vec{Z}\prime$ and the elevation of origin of each landrace beyond what would be expected due to neutral drift using the methods described in Berg and Coop (2014). We transformed both $\vec{Z}\prime$ and the mean-centered vector of elevations for each landrace by the Cholesky decomposition of the kinship matrix (*C*) using the following equation (shown here for $\vec{Z}\prime$)

(9) $$\vec{X}=\frac{1}{\sqrt{2V_{A}}}C^{-1}(\vec{Z}\prime )$$

We estimate *V_A_* using the allele frequencies and effect sizes of the GWAS loci, using the following formula. If $\bar{p_{m}}$ is the allele frequency of the *m*th locus across all individuals and *α_m_* is the effect size of that locus,

(10) $$V_{A}=\sum_{m=1}^{M}\alpha_{m}^{2}\bar{p_{m}}(1-\bar{p_{m}})$$

We then test for a linear relationship between $\vec{X}$ and the similarly transformed vector of elevations using the lm function in R (R Core Team 2018) and control for the 29 tests done using Qvalue (Storey and Tibshirani 2003).

## Supplementary Material

Supplementary data are available at *Molecular Biology and Evolution* online.

## Supplementary Material

msab119_Supplementary_Data
