# Supplementary material for: Molecular Parallelism Underlies Convergent Highland Adaptation of Maize Landraces
Source: Mol Biol Evol. 2021 Apr 27;38(9):3567–80. doi: 10.1093/molbev/msab119 (PMC8382895; doi:10.1093/molbev/msab119)
Supplement: msab119_Supplementary_Data [file msab119_supplementary_data.zip › polygenic.pdf]

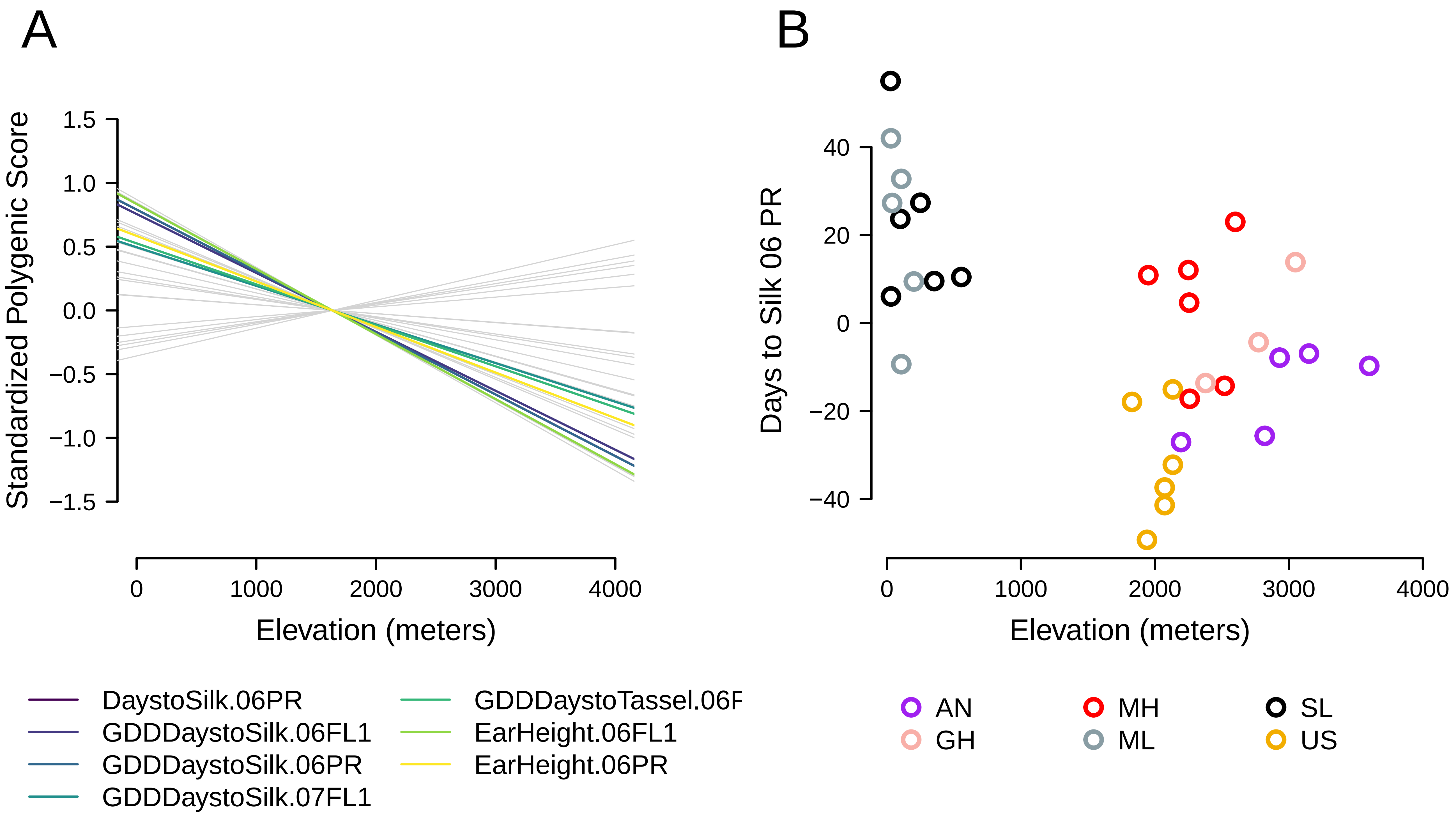

A
B
Standardized Polygenic Score
1.5
1.0
0.5
0.0
−0.5
−1.0
−1.5
0
1000
2000
3000
4000
Elevation (meters)
DaystoSilk.06PR
GDDDaystoSilk.06FL1
GDDDaystoSilk.06PR
GDDDaystoSilk.07FL1
GDDDaystoTassel.06F
EarHeight.06FL1
EarHeight.06PR
Days to Silk 06 PR
40
20
0
−20
−40
0
1000
2000
3000
4000
Elevation (meters)
AN
GH
MH
ML
SL
US
