# Supplementary figures and images for: Molecular Parallelism Underlies Convergent Highland Adaptation of Maize Landraces
Source: Mol Biol Evol. 2021 Apr 27;38(9):3567–80. doi: 10.1093/molbev/msab119 (PMC8382895; doi:10.1093/molbev/msab119)

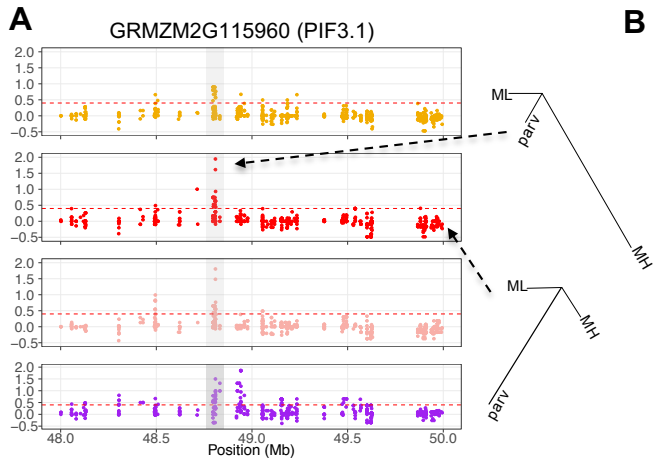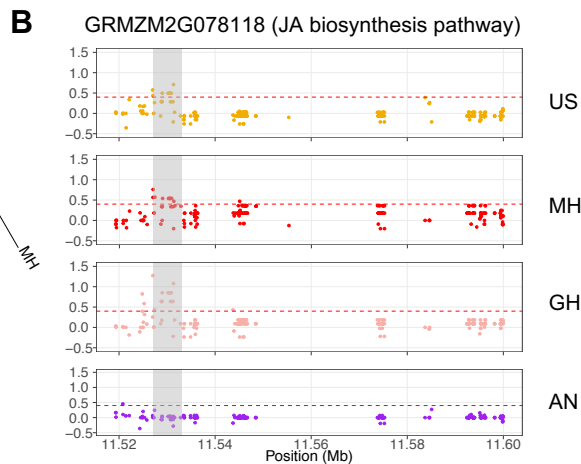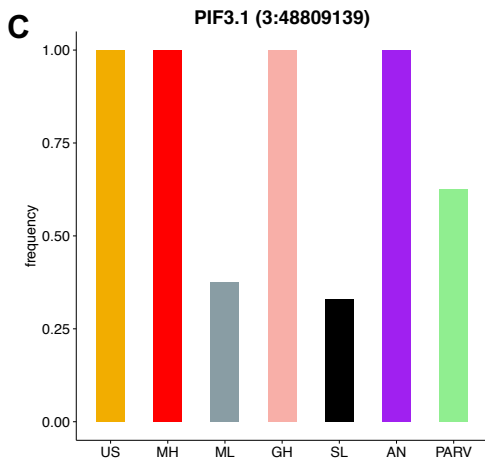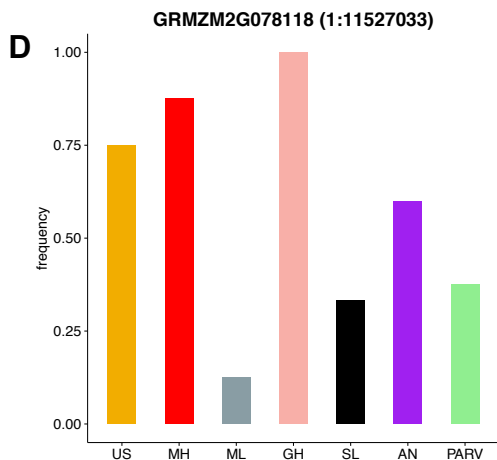

Supplement: msab119_Supplementary_Data [file msab119_supplementary_data.zip › Figure2new.pdf]

**A**

SNPtype anti co

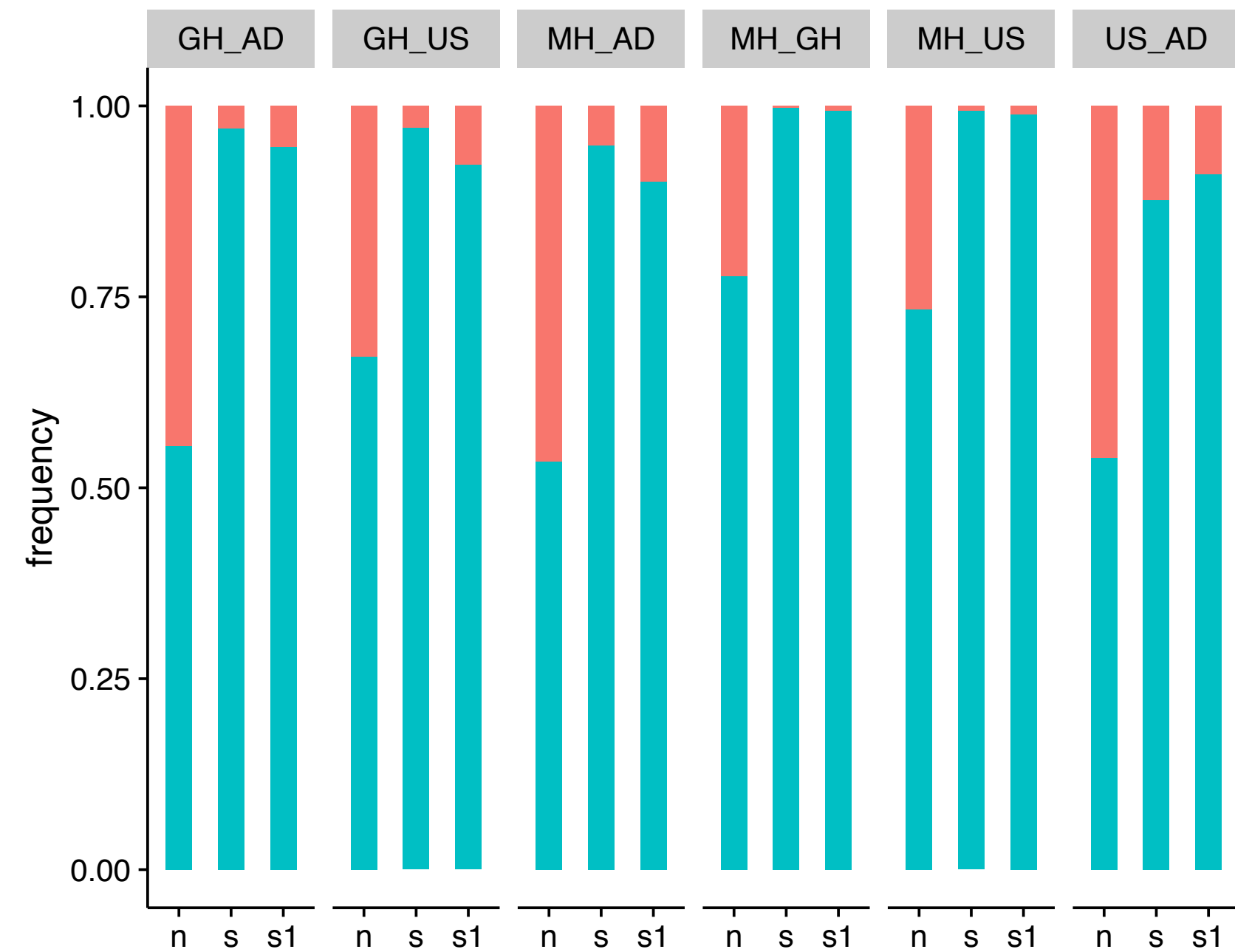**B**

Number of the best fit model

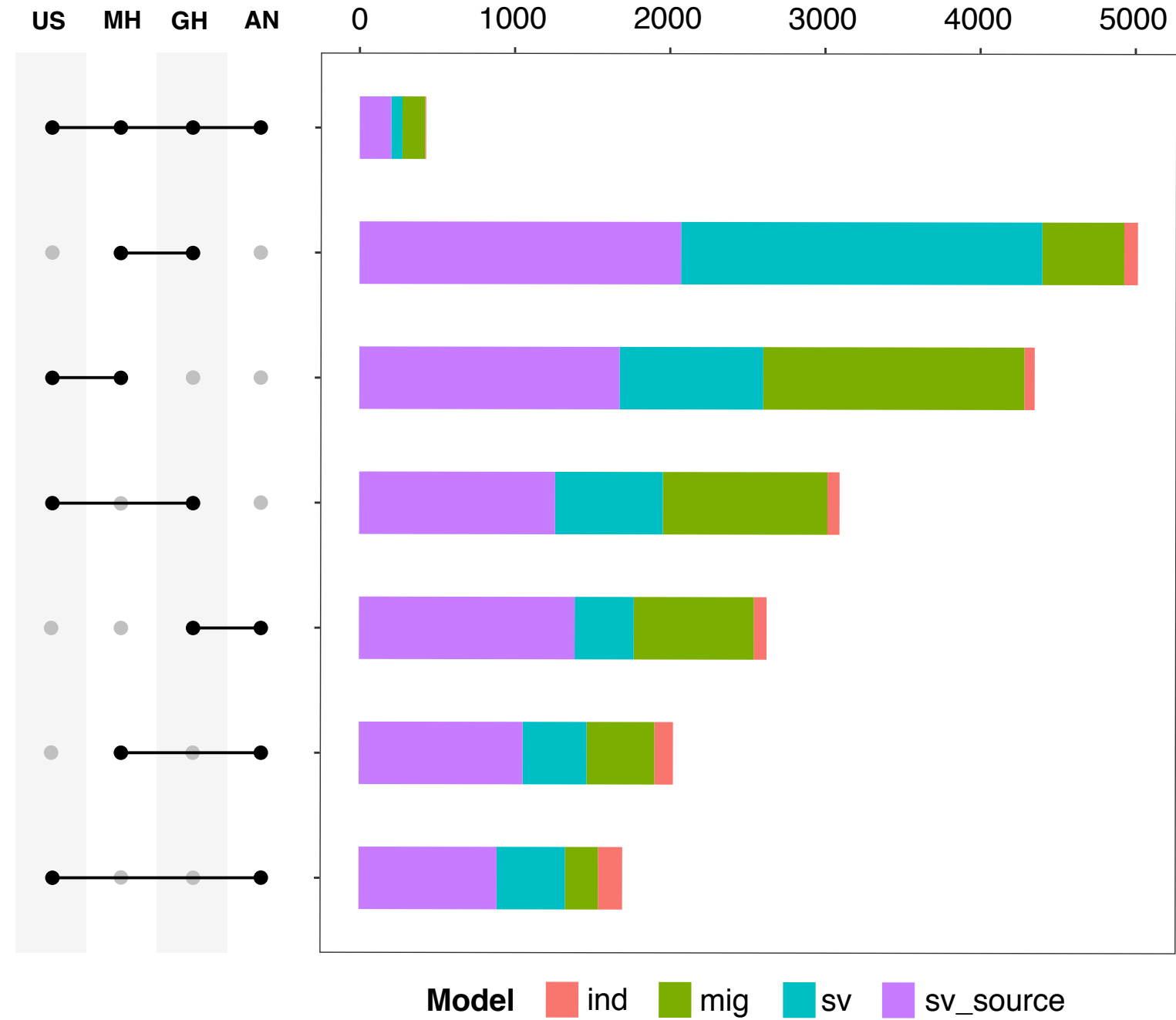

Supplement: msab119_Supplementary_Data [file msab119_supplementary_data.zip › Figure3new.pdf]

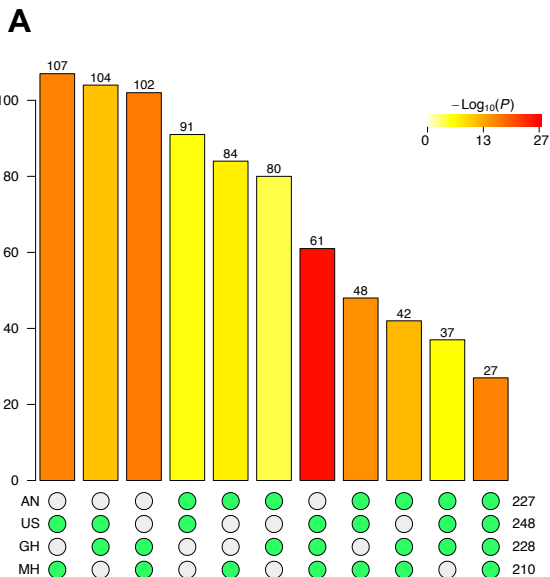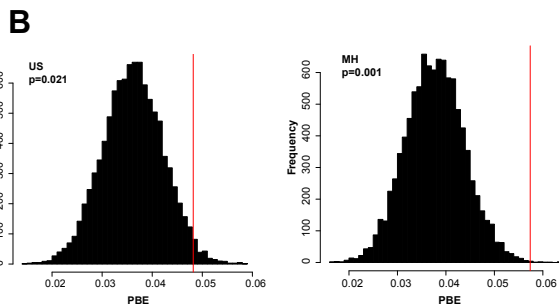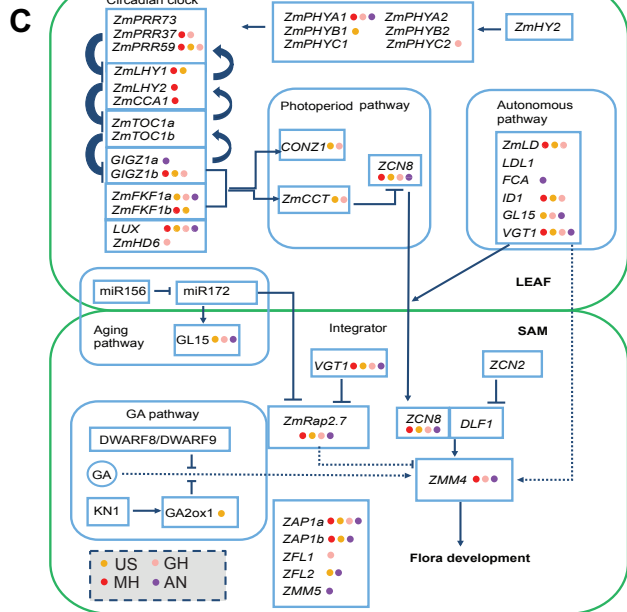

Supplement: msab119_Supplementary_Data [file msab119_supplementary_data.zip › Figure4.pdf]

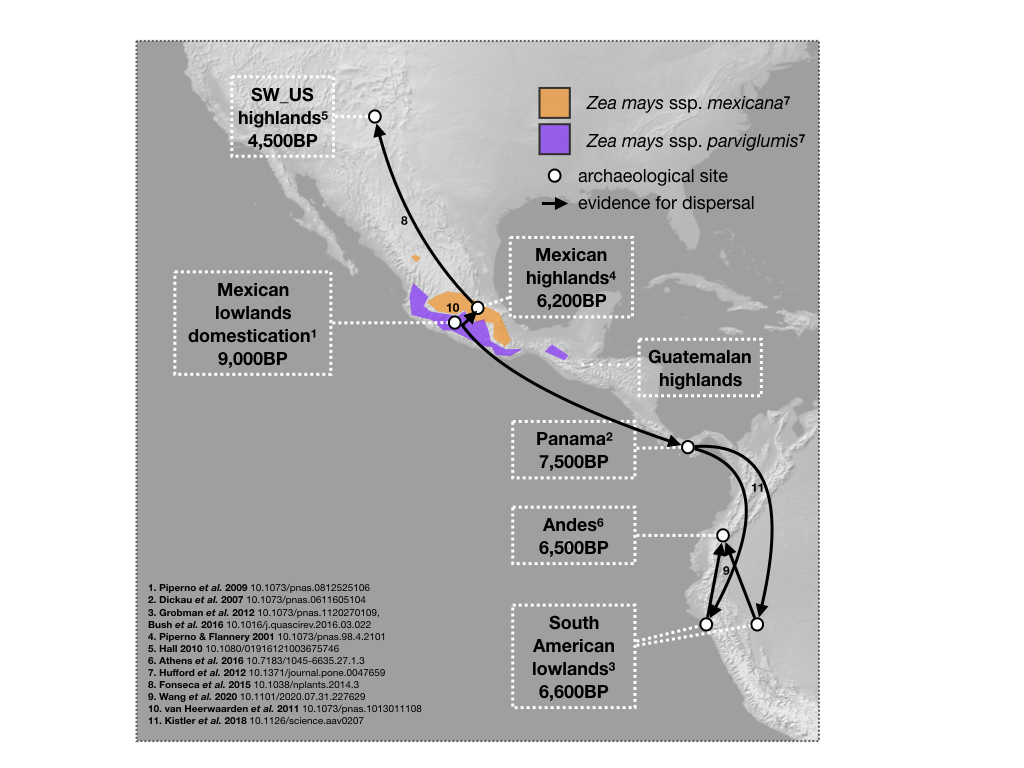

Supplement: msab119_Supplementary_Data [file msab119_supplementary_data.zip › map.jpeg]

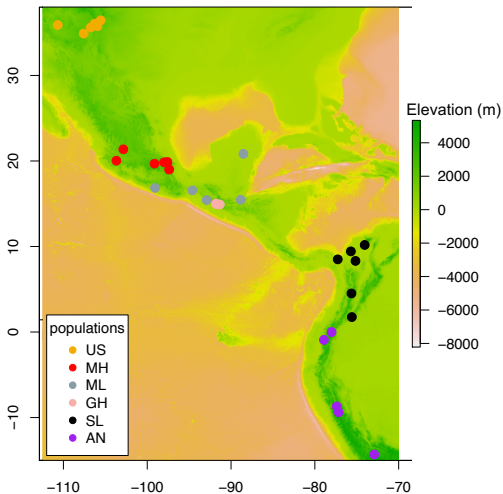

Supplement: msab119_Supplementary_Data [file msab119_supplementary_data.zip › sampling.pdf]

**A**

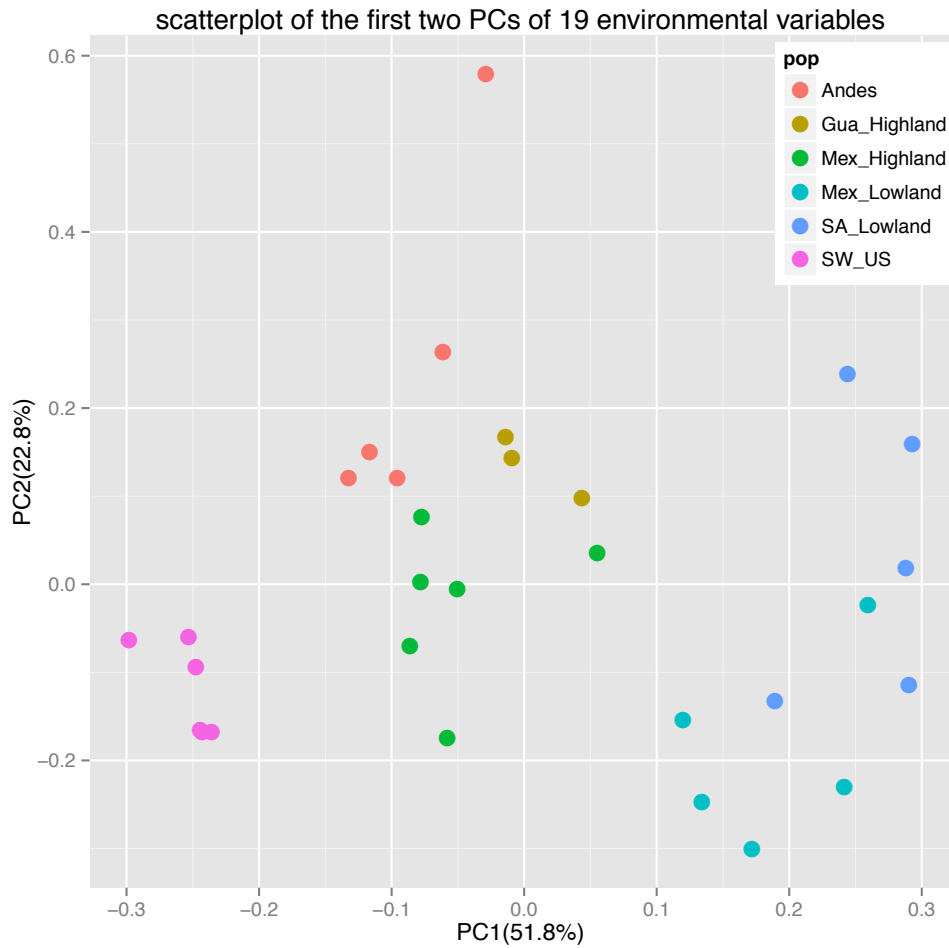

**B**

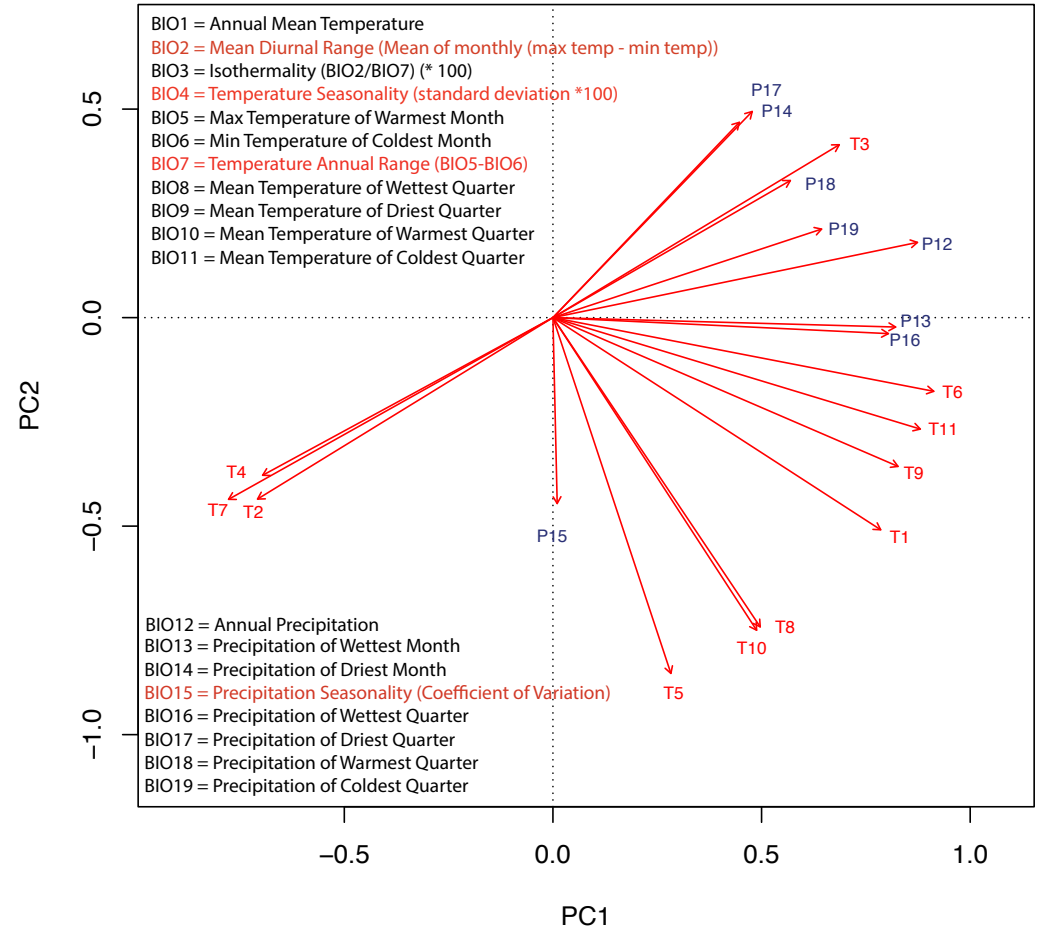

Supplement: msab119_Supplementary_Data [file msab119_supplementary_data.zip › sFig1.pdf]

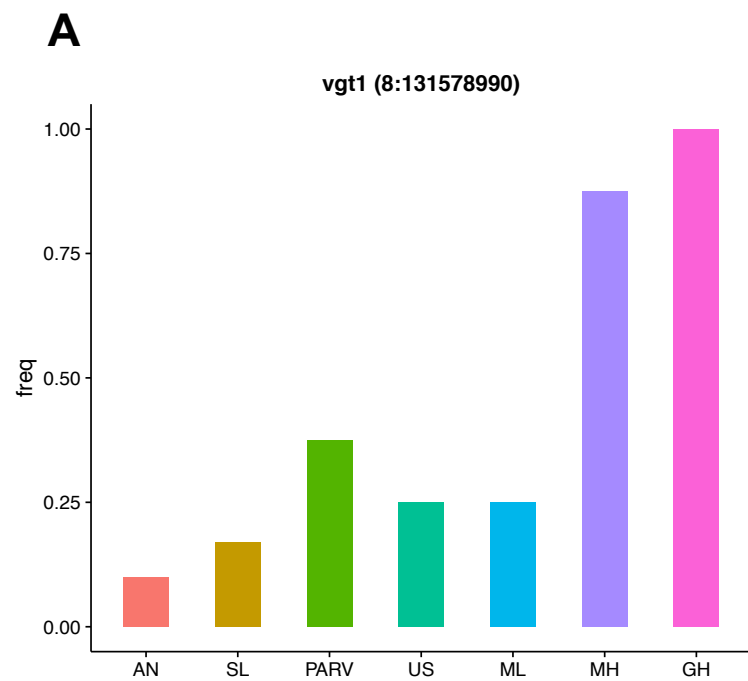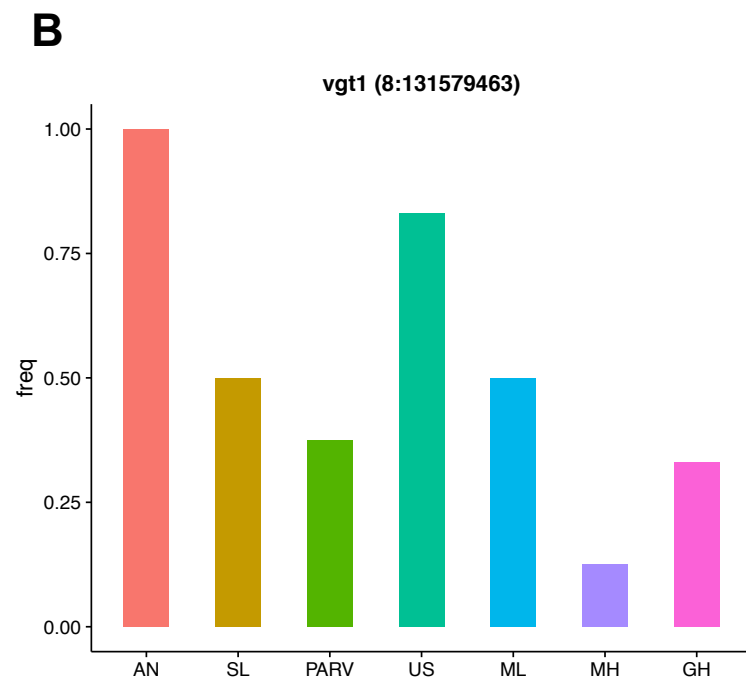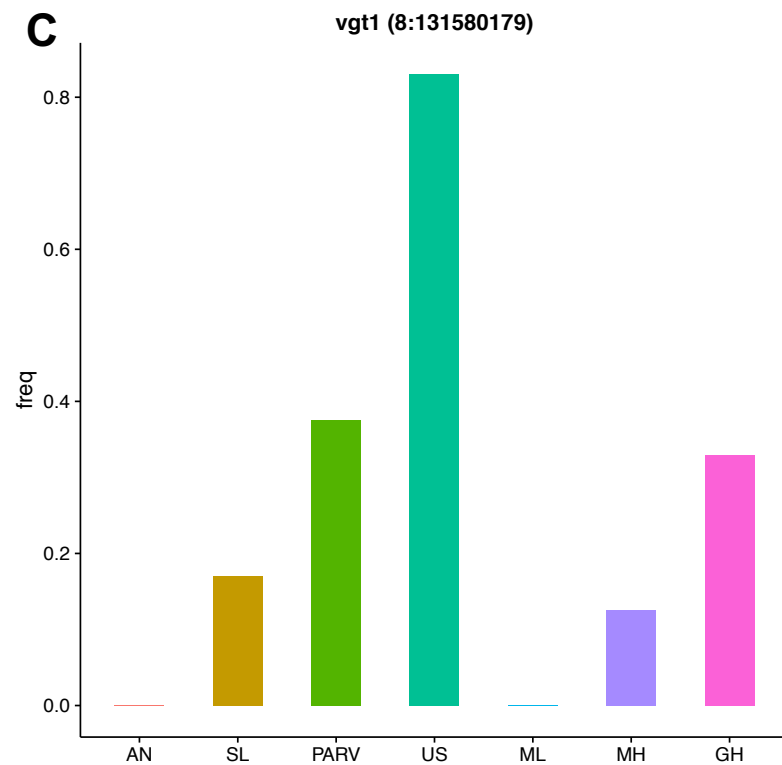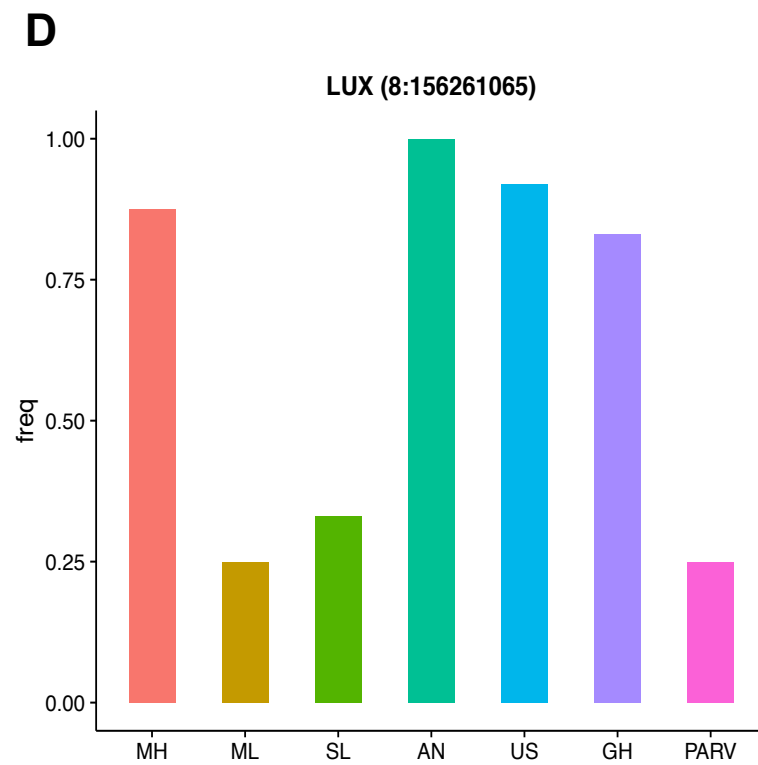

Supplement: msab119_Supplementary_Data [file msab119_supplementary_data.zip › sFig10.pdf]

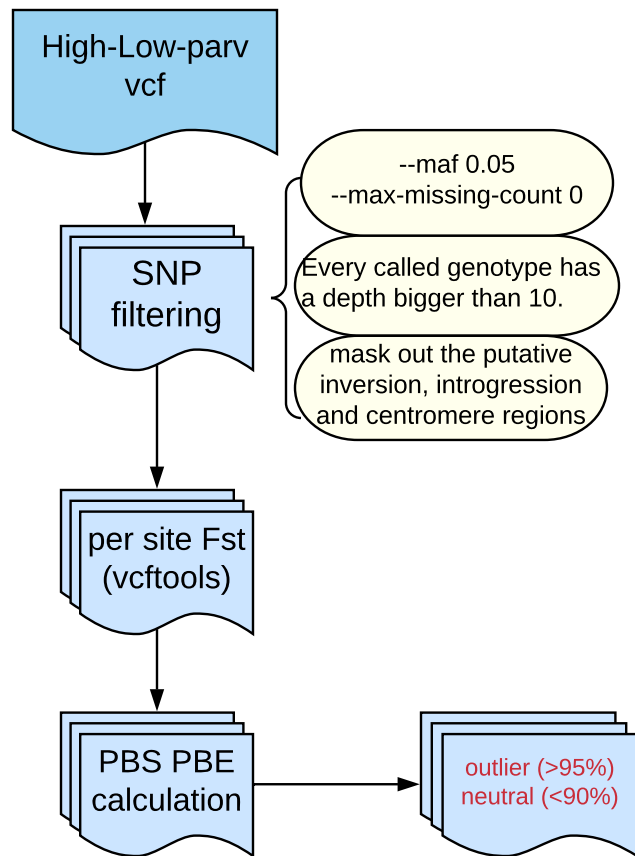

Supplement: msab119_Supplementary_Data [file msab119_supplementary_data.zip › sFig11PBEcalcFlowChart.pdf]

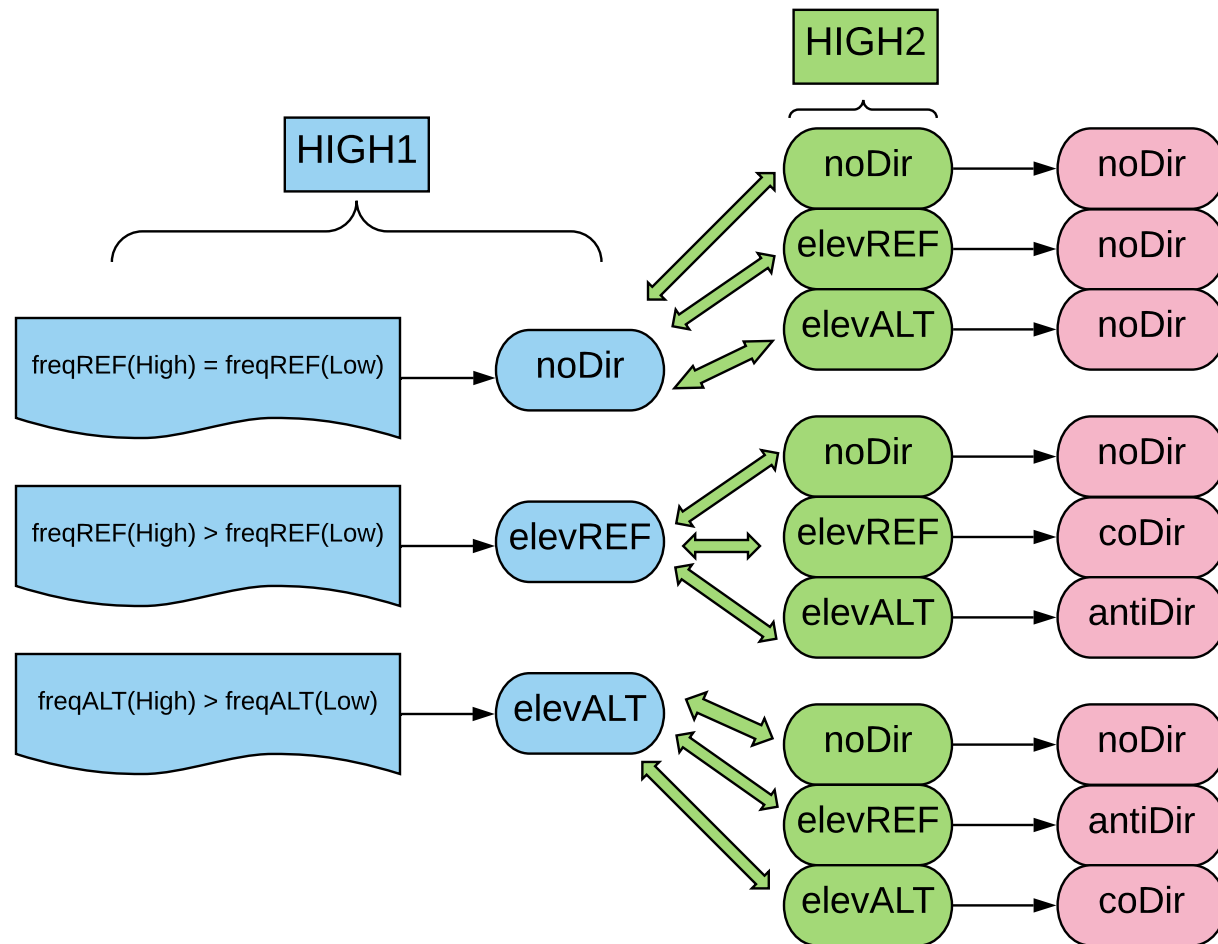

Supplement: msab119_Supplementary_Data [file msab119_supplementary_data.zip › sFig12coAntiDirFlowchart.pdf]

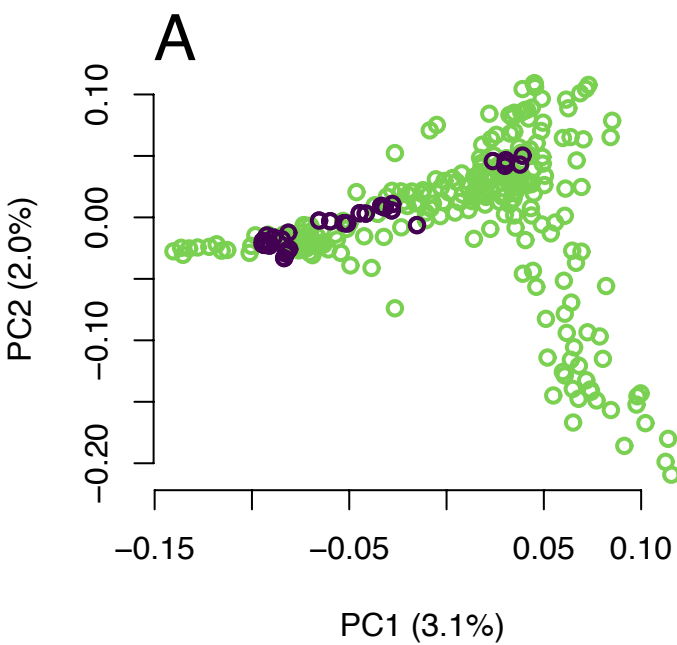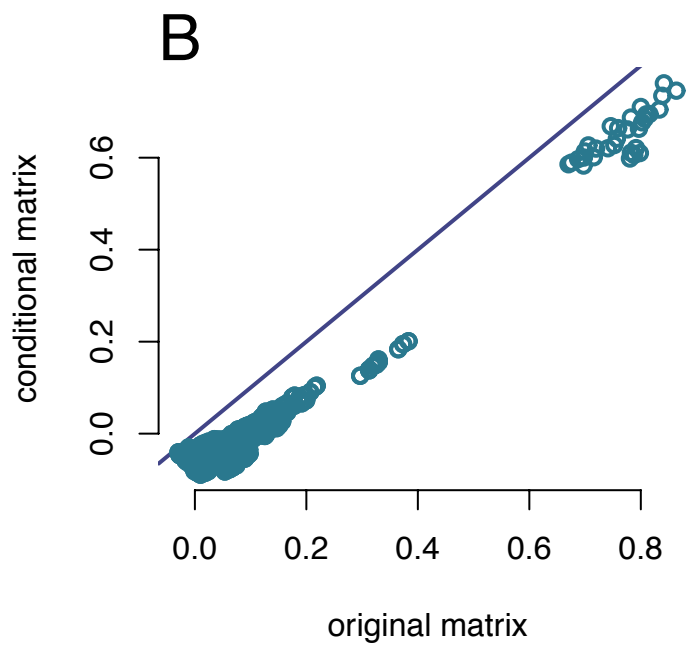

Supplement: msab119_Supplementary_Data [file msab119_supplementary_data.zip › sFig13sharedstructure.pdf]

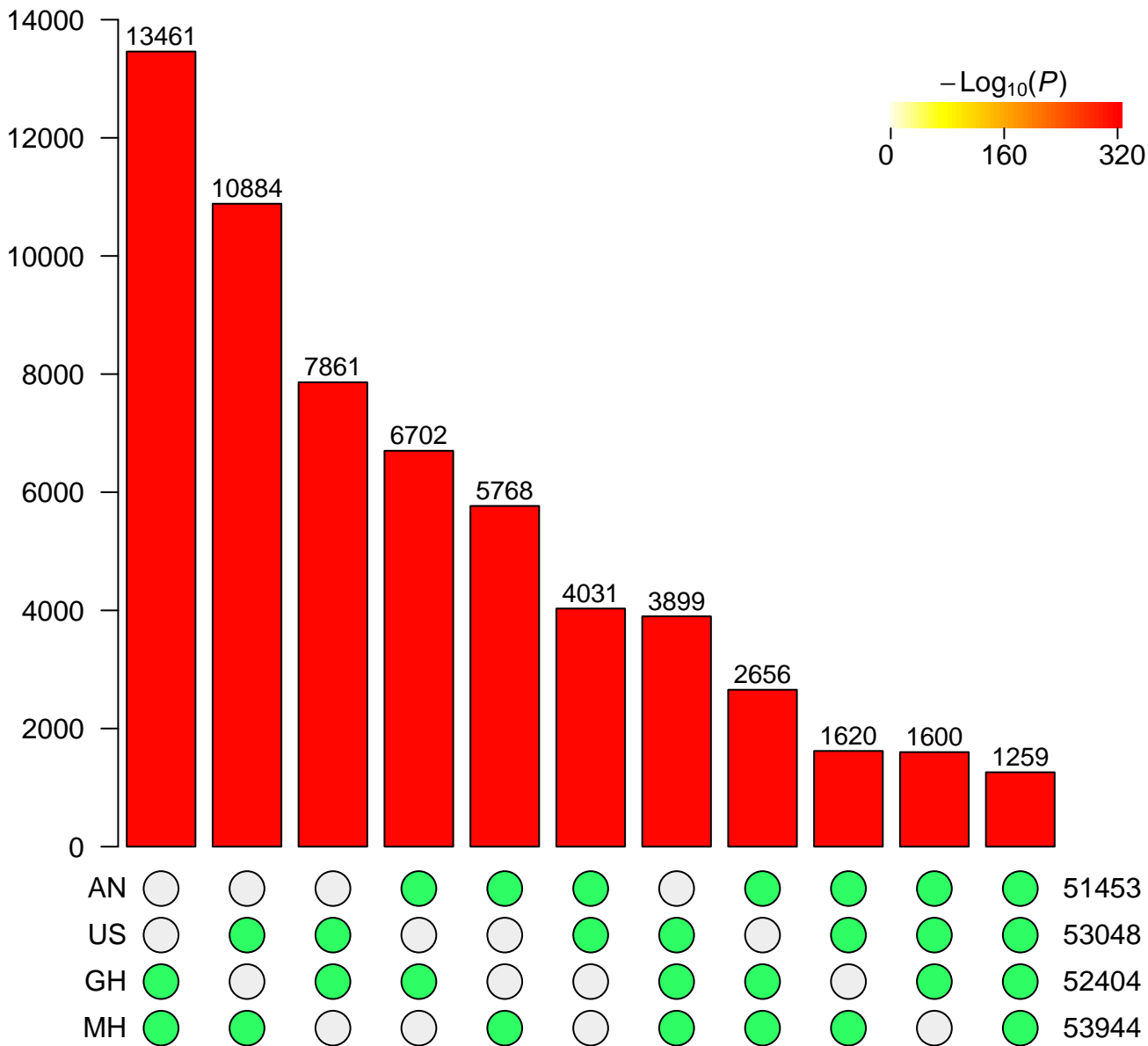

Supplement: msab119_Supplementary_Data [file msab119_supplementary_data.zip › sFig2SNPintersection.pdf]

Number of elements

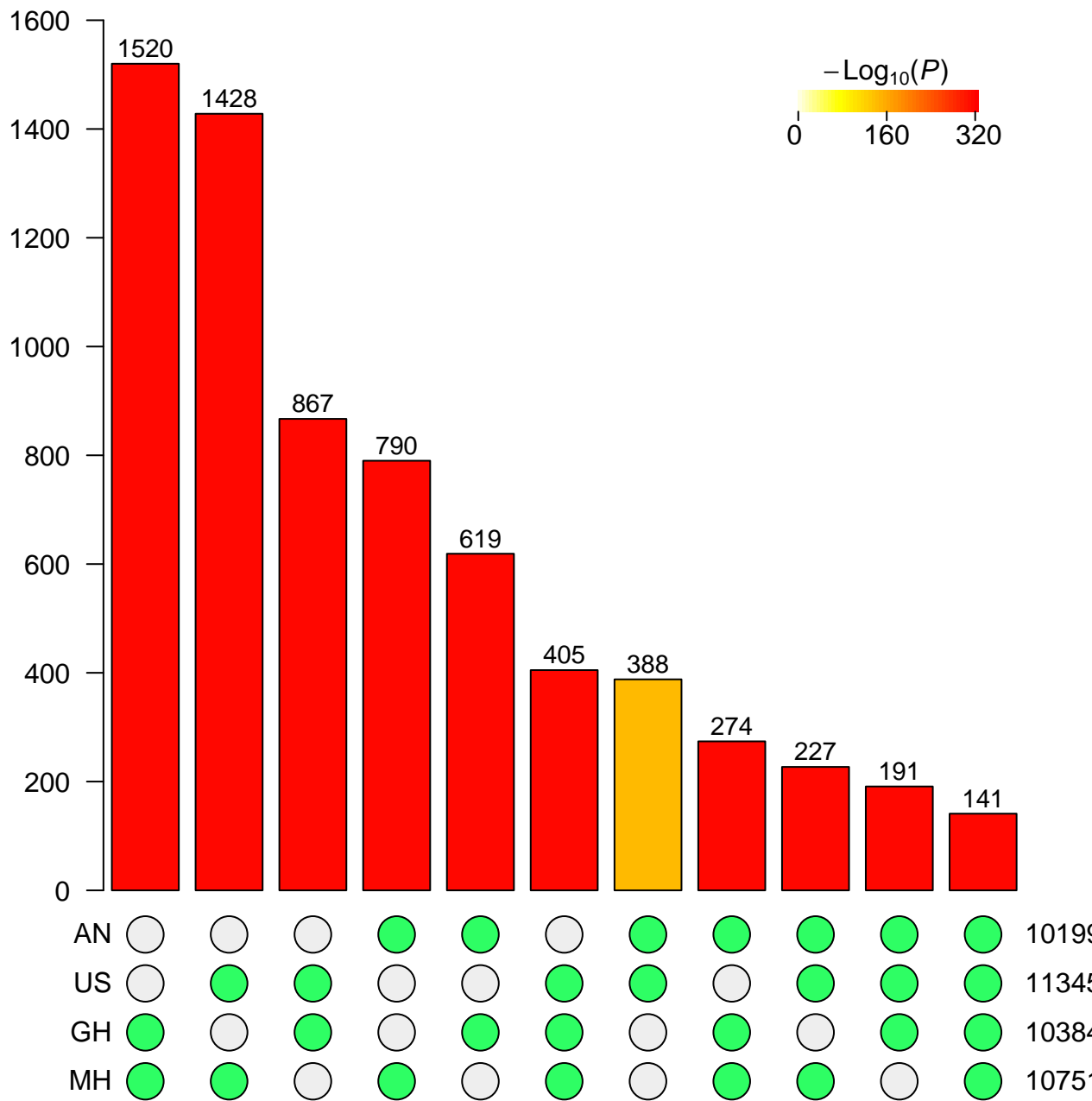

Supplement: msab119_Supplementary_Data [file msab119_supplementary_data.zip › sFig3SNPintersection1.pdf]

Number of elements

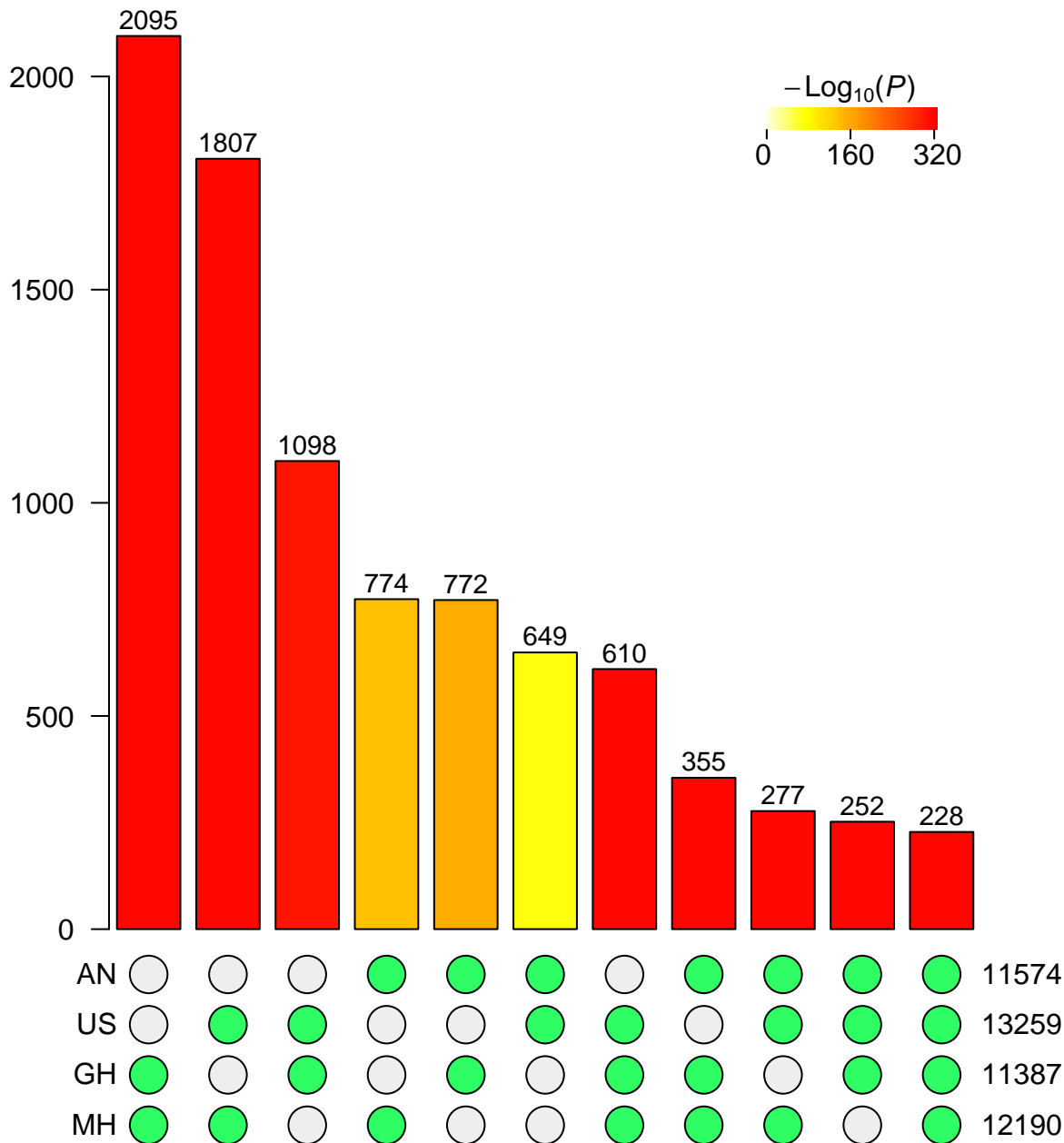

Supplement: msab119_Supplementary_Data [file msab119_supplementary_data.zip › sFig3SNPintersection2.pdf]

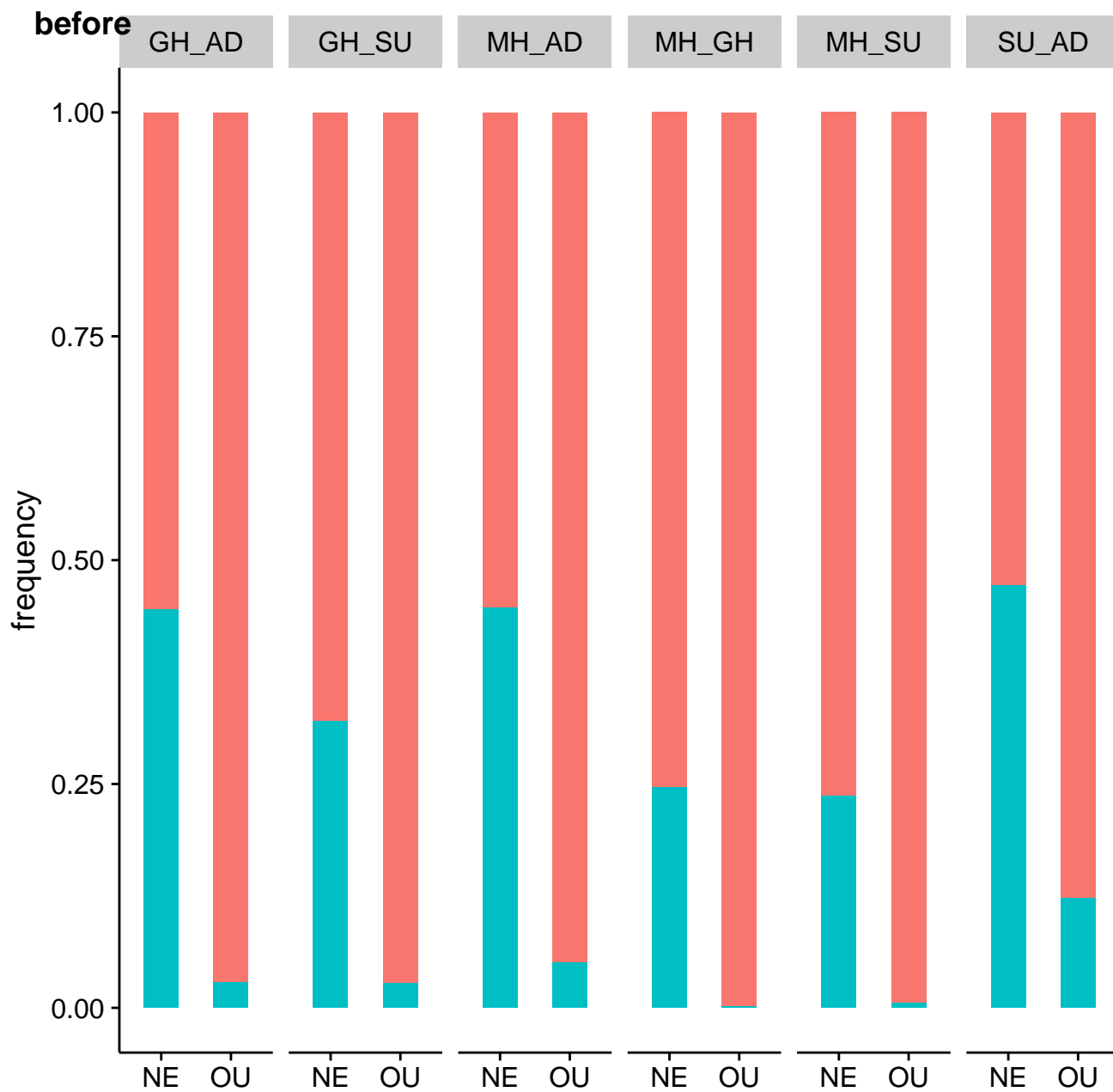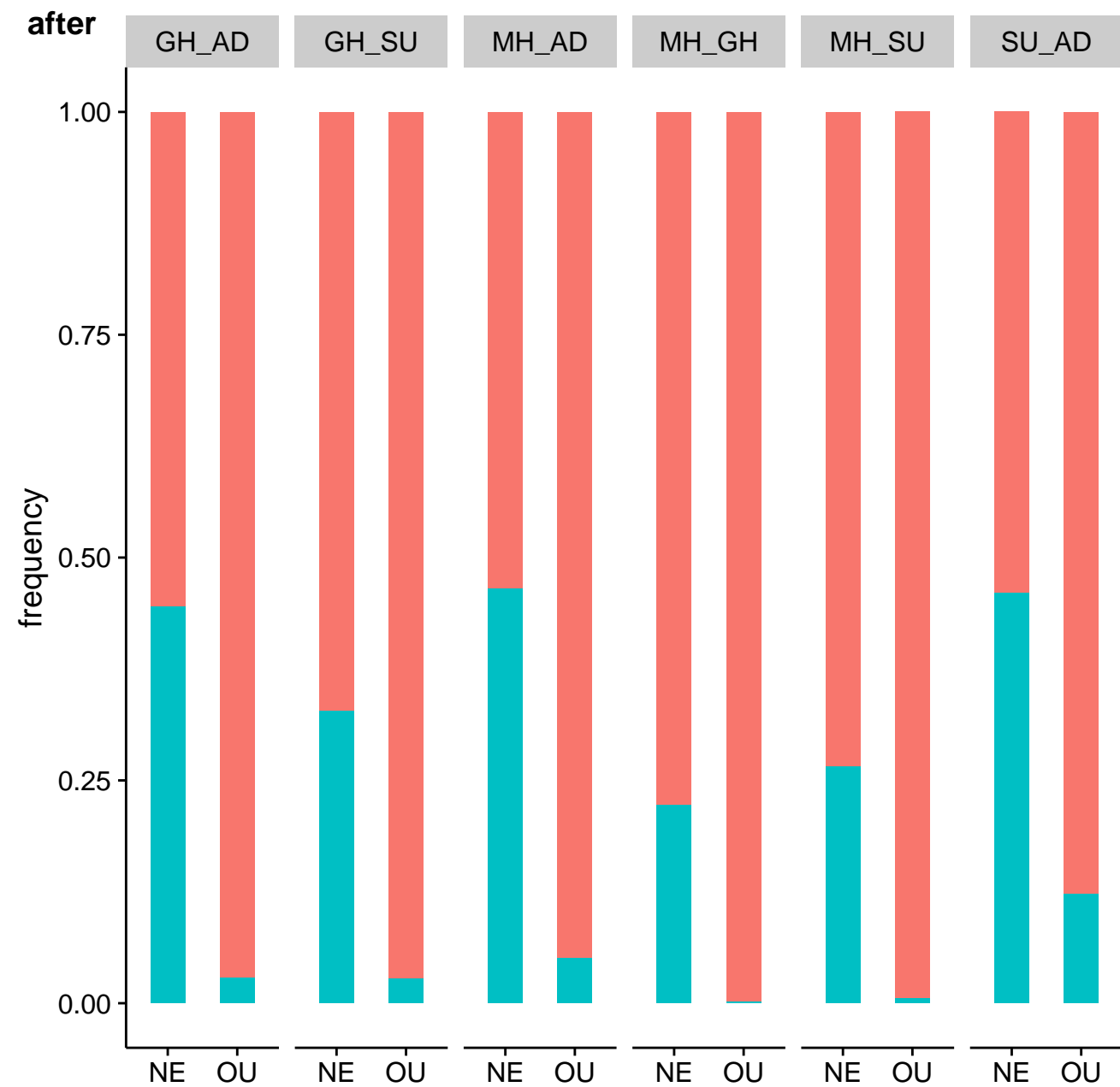

Supplement: msab119_Supplementary_Data [file msab119_supplementary_data.zip › sFig4coAnti2dsfs.pdf]

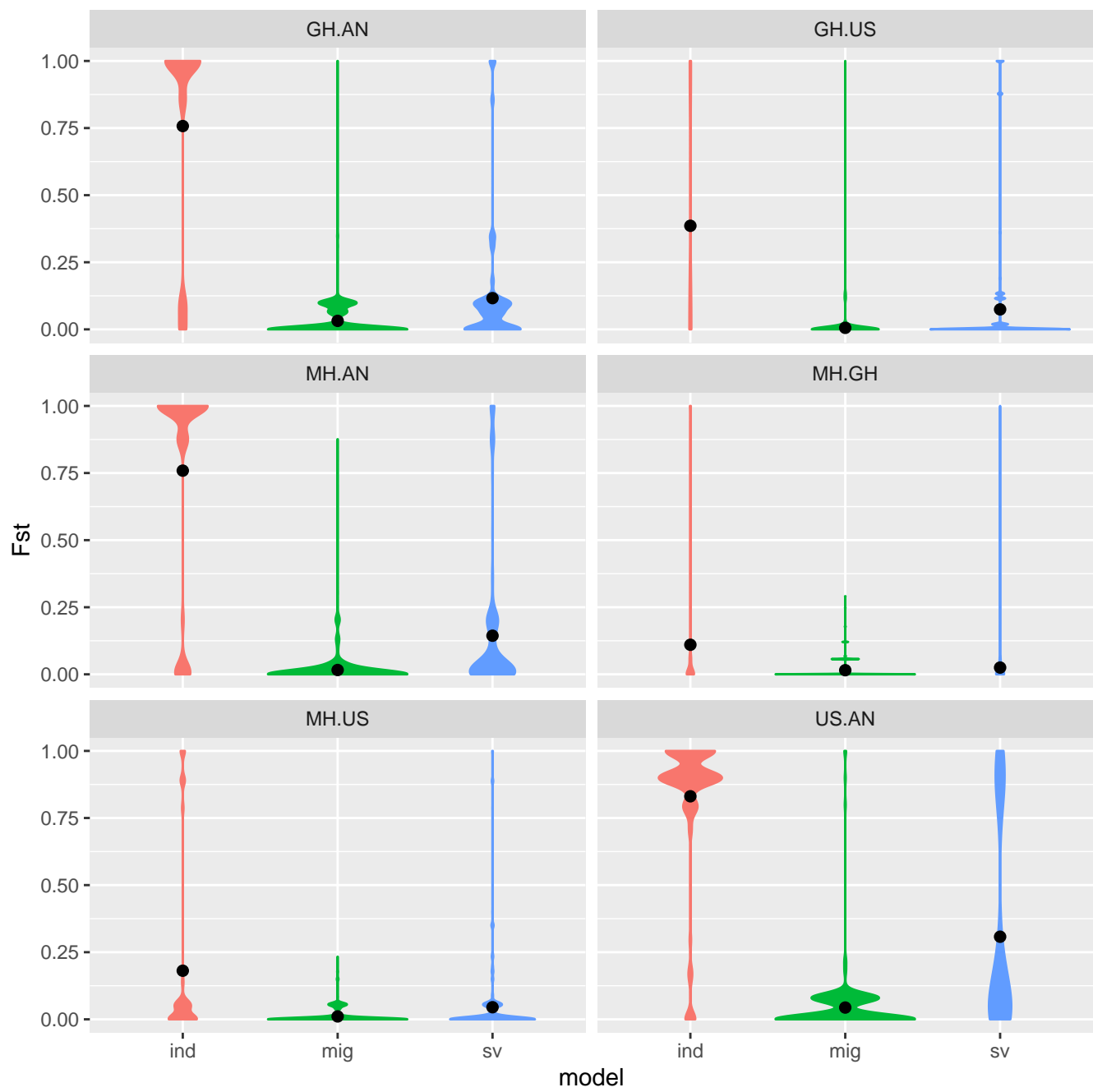

Supplement: msab119_Supplementary_Data [file msab119_supplementary_data.zip › sFig5FstModel.pdf]

fdM

GH.AN

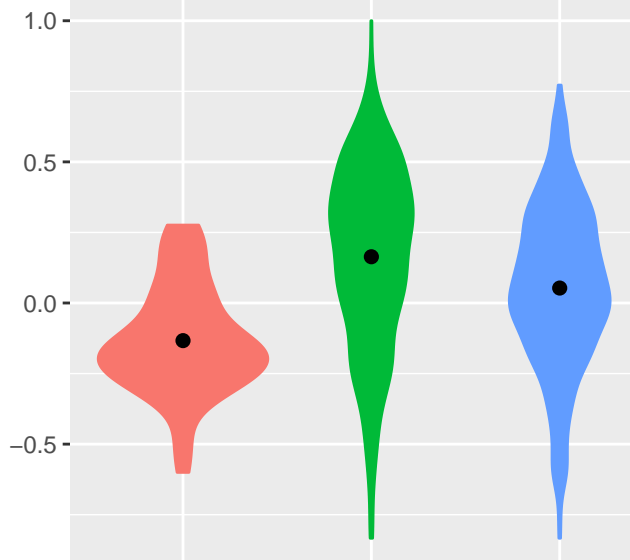

MH.AN

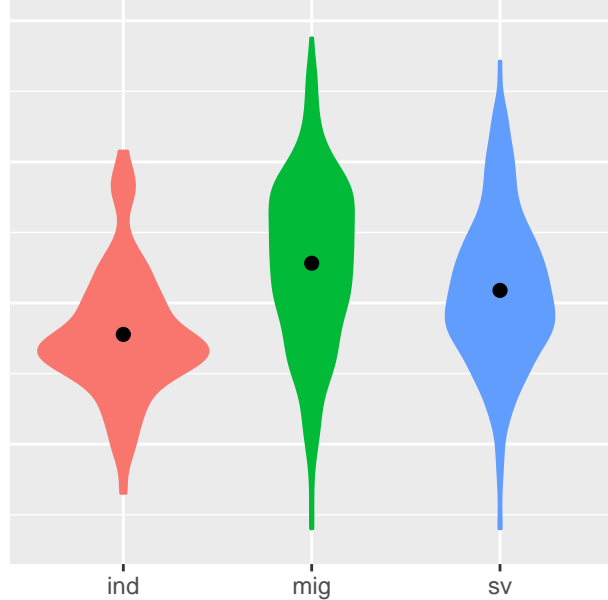

US.AN

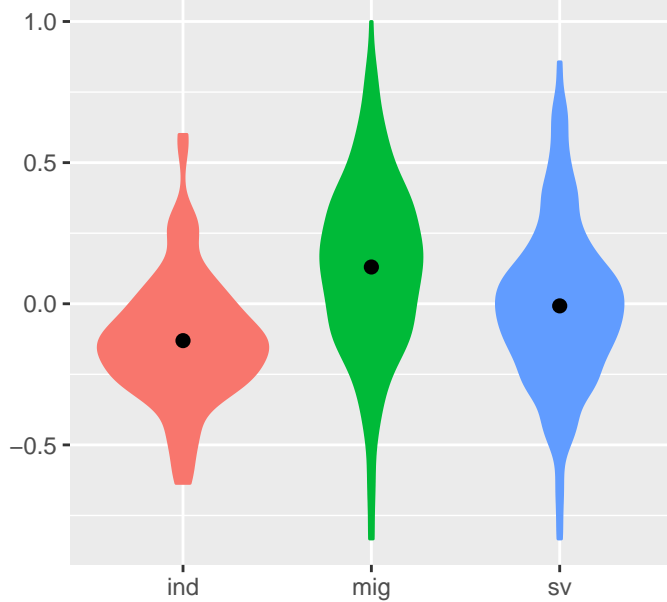

model

Supplement: msab119_Supplementary_Data [file msab119_supplementary_data.zip › sFig6fdMmodel.pdf]

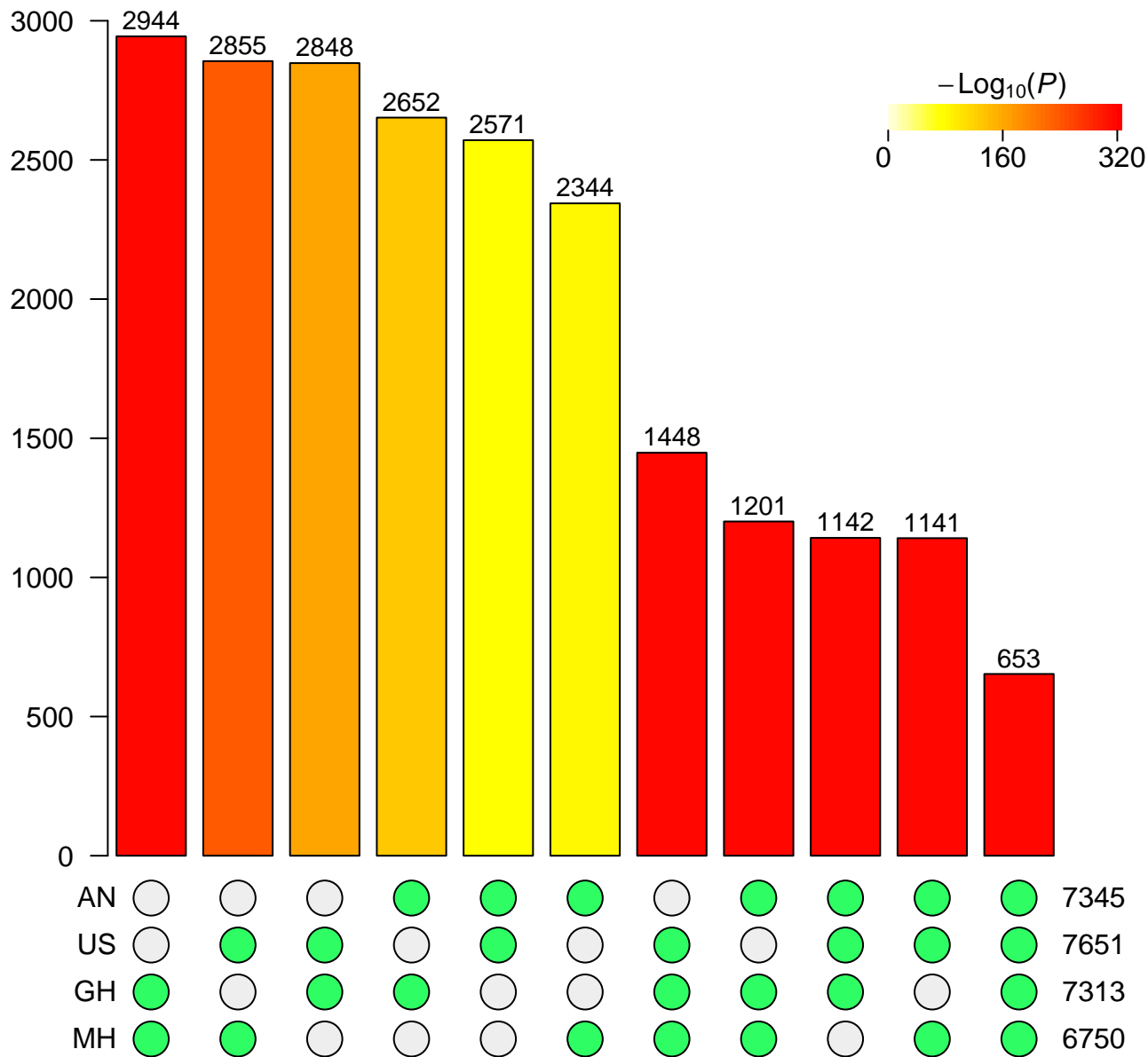

Supplement: msab119_Supplementary_Data [file msab119_supplementary_data.zip › sFig7GeneIntersection5.pdf]

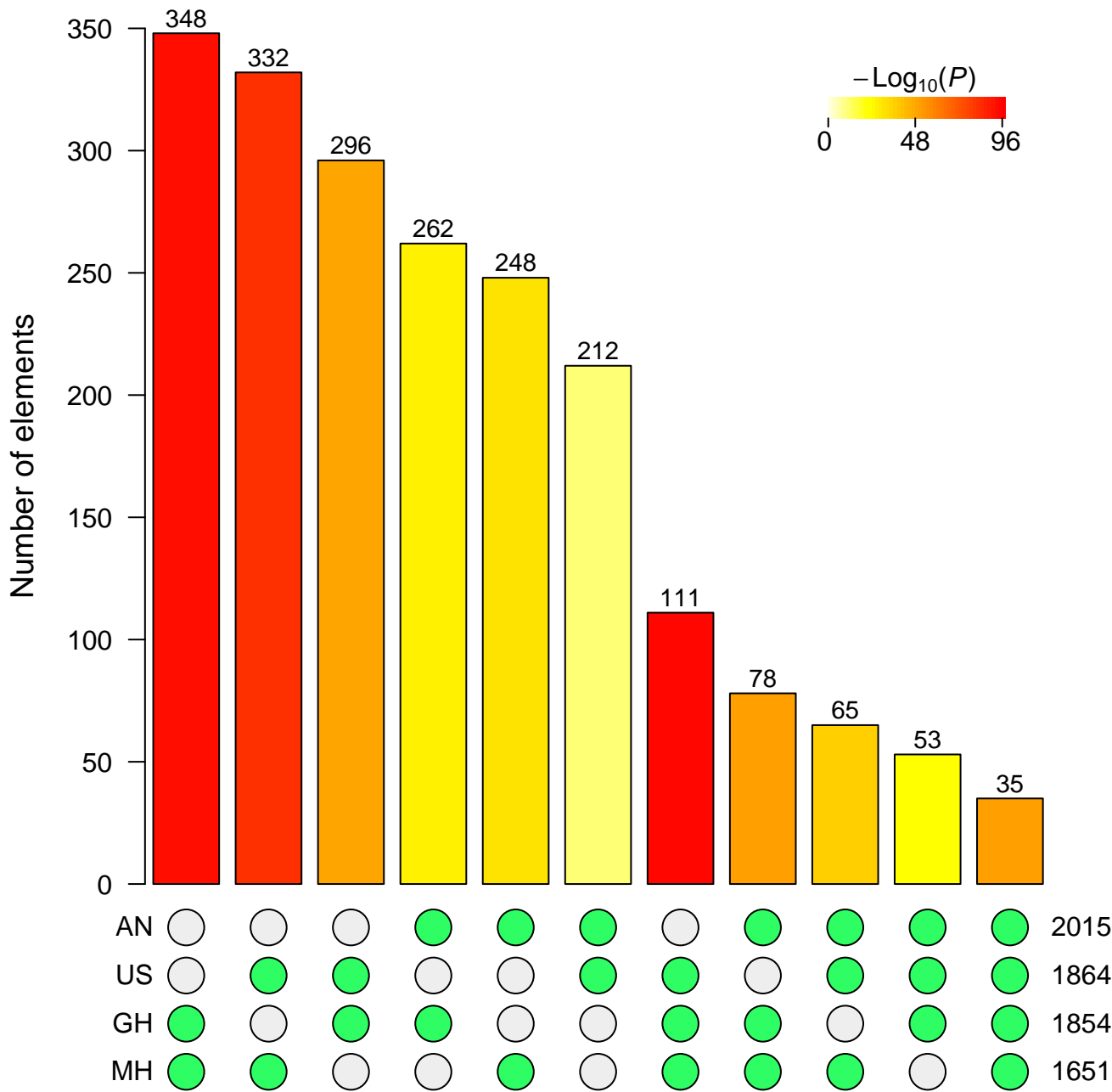

Supplement: msab119_Supplementary_Data [file msab119_supplementary_data.zip › sFig8GeneIntersection1.pdf]

Number of elements

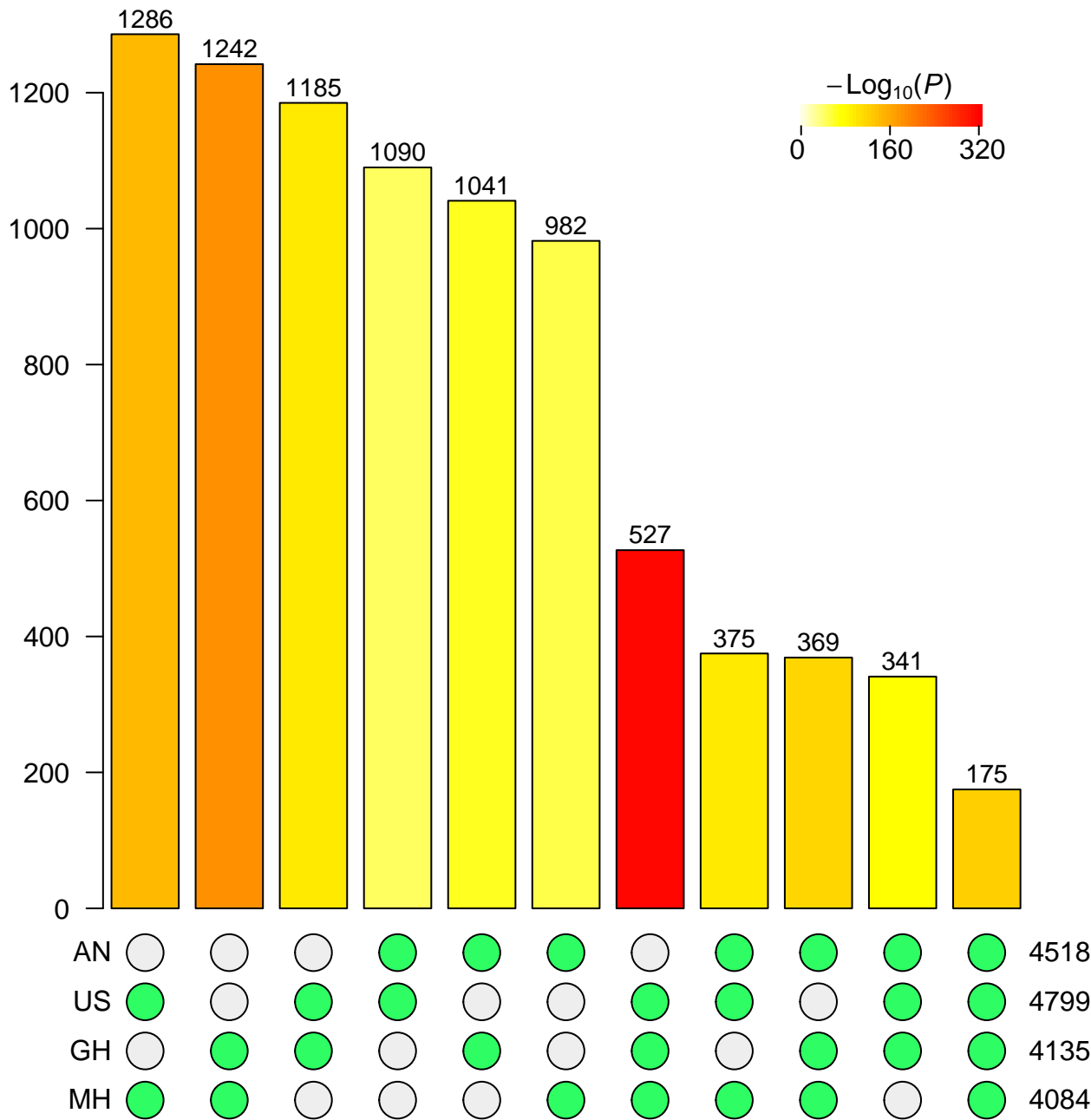

Supplement: msab119_Supplementary_Data [file msab119_supplementary_data.zip › sFig8GeneIntersectionThinned.pdf]

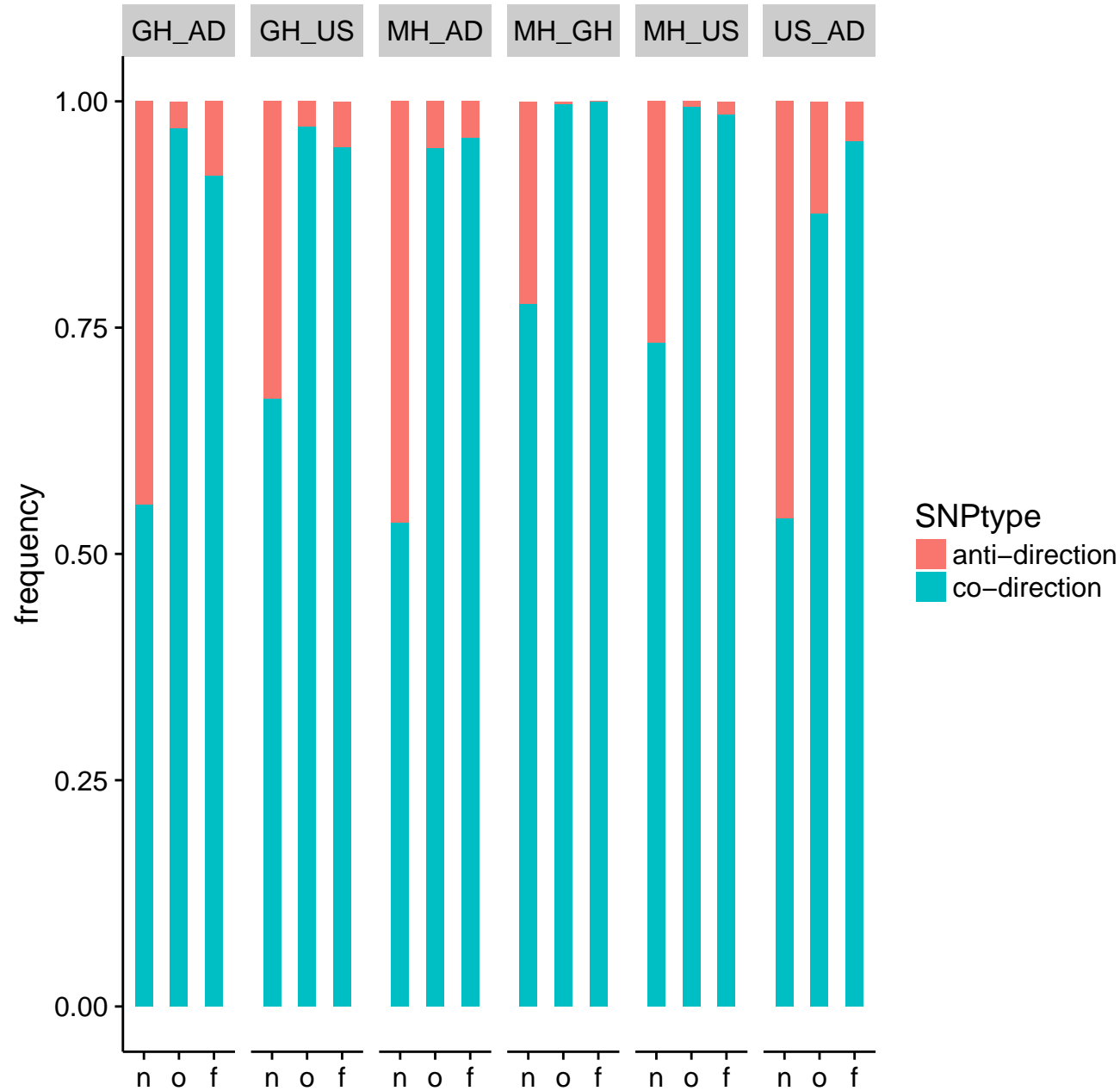

Supplement: msab119_Supplementary_Data [file msab119_supplementary_data.zip › sFig9CoAntiSummary.pdf]
